# Supplementary material for: Stomatal CO2/bicarbonate sensor consists of two interacting protein kinases, Raf-like HT1 and non-kinase-activity requiring MPK12/MPK4
Source: Sci Adv. 2022 Dec 7;8(49):eabq6161. doi: 10.1126/sciadv.abq6161 (PMC9728965; doi:10.1126/sciadv.abq6161)
Supplement: 20221207-1 [file sciadv.abq6161.v1.pdf]

PLANT SCIENCES

Stomatal CO_2/bicarbonate sensor consists of two interacting protein kinases, Raf-like HT1 and non-kinase-activity requiring MPK12/MPK4

Copyright © 2022 The Authors, some rights reserved; exclusive licensee American Association for the Advancement of Science. No claim to original U.S. Government Works. Distributed under a Creative Commons Attribution License 4.0 (CC BY).

Yohei Takahashi[1]*†, Krystal C. Bosmans[1], Po-Kai Hsu[1], Karnelia Paul[1], Christian Seitz[2], Chung-Yueh Yeh[3], Yuh-Shuh Wang[3], Dmitry Yarmolinsky[3], Maija Sierla[4], Triin Vahisalu[4], J. Andrew McCammon[2,5], Jaakko Kangasjärvi[4], Li Zhang[1], Hannes Kollist[3], Thien Trac[1], Julian I. Schroeder[1]*

The continuing rise in the atmospheric carbon dioxide (CO_2) concentration causes stomatal closing, thus critically affecting transpirational water loss, photosynthesis, and plant growth. However, the primary CO_2 sensor remains unknown. Here, we show that elevated CO_2 triggers interaction of the MAP kinases MPK4/MPK12 with the HT1 protein kinase, thus inhibiting HT1 kinase activity. At low CO_2, HT1 phosphorylates and activates the downstream negatively regulating CBC1 kinase. Physiologically relevant HT1-mediated phosphorylation sites in CBC1 are identified. In a genetic screen, we identify dominant active HT1 mutants that cause insensitivity to elevated CO_2. Dominant HT1 mutants abrogate the CO_2/bicarbonate-induced MPK4/12-HT1 interaction and HT1 inhibition, which may be explained by a structural AlphaFold2- and Gaussian-accelerated dynamics-generated model. Unexpectedly, MAP kinase activity is not required for CO_2 sensor function and CO_2-triggered HT1 inhibition and stomatal closing. The presented findings reveal that MPK4/12 and HT1 together constitute the long-sought primary stomatal CO_2/bicarbonate sensor upstream of the CBC1 kinase in plants.

INTRODUCTION

Plant stomata open and close rapidly in response to changing environmental conditions, thereby regulating gas exchange between plants and the atmosphere. CO_2 influx into leaves from the atmosphere is essential for plant photosynthesis. Stomatal conductance is regulated by dynamic and rapid stomatal movements (*1*–*5*). Plants sense diurnal dark/light-induced changes in the CO_2 concentration (C_i) in the intercellular air spaces of leaves, thus causing opening and closing of stomata (*5*). Furthermore, the continuing rise in the atmospheric CO_2 concentration is narrowing stomatal pores globally (*1*, *6*). Elevation in the leaf CO_2 concentration causes rapid stomatal closing, thus reducing transpirational water loss from plants. Conversely, in response to low CO_2, stomata open and increase stomatal conductance. The stomatal CO_2 response, therefore, is critical for plant growth and regulates the water use efficiency of plants. CO_2-induced stomatal movements in dicots and monocots require catalytic carbonic anhydrase activity (*7*, *8*). These carbonic anhydrases accelerate the catalysis of CO_2 entering through the plasma membrane to lipid membrane impermeable bicarbonate ions (HCO_3^-) and protons. Data indicate that the accumulated bicarbonate ions are an important intracellular messenger in guard cells that mediate stomatal closure (*7*, *9*–*11*). However, the primary CO_2/bicarbonate sensor has remained elusive. This sensor is required for regulation of early protein phosphorylation events that drive CO_2-regulated stomatal movements (*9*, *12*–*14*).

Using infrared thermal imaging, a CO_2-insensitive *Arabidopsis* mutant was isolated, and the causative gene was identified as a Raf-like protein kinase named high leaf temperature 1 (HT1), suggesting an important role for protein phosphorylation in CO_2-induced stomatal movements (*12*). Recessive *ht1-2* mutant stomata show a constitutively high CO_2-like closed stomatal phenotype regardless of the CO_2 concentration but respond to blue light and the plant hormone abscisic acid (*12*). Furthermore, other Raf-like protein kinases CONVERGENCE OF BLUE LIGHT AND CO_2 1 (CBC1) and CBC2 are essential for the stomatal CO_2 response (*13*). Because *cbc1 cbc2* double mutants show closed stomata similar to the *ht1-2* mutant, the HT1 and CBC kinases are considered to be negative regulators of high CO_2-induced stomatal closure, but the underlying CO_2 regulation mechanisms remain unknown.

Conversely, double-mutant alleles in the *Arabidopsis* mitogen-activated protein kinase4 (MPK4) and MPK12 mitogen-activated protein (MAP) kinases show constitutively open stomata and insensitivity to high CO_2 concentrations but an intact abscisic acid response, suggesting that these MAP kinases are redundant positive regulators of early CO_2 signal transduction in guard cells (*14*). However, the CO_2 sensor remains unknown, and the signaling network mechanisms remain unclear. Here, we reveal that the CO_2 sensor consists of the protein complex of MPK4/12 with the HT1 protein kinase. Elevated CO_2/bicarbonate causes a direct interaction of MPK4 and MPK12 with HT1, thereby directly inhibiting HT1 activity and downstream CBC1 activity. Moreover, we unexpectedly find that MAP kinase activity is not required for CO_2 sensor signaling and CO_2-regulated stomatal movements in vivo.

[1]School of Biological Sciences, Cell and Developmental Biology Department, University of California San Diego, La Jolla, CA 92093-0116, USA. [2]Department of Chemistry and Biochemistry, University of California San Diego, La Jolla, CA 92093, USA. [3]Institute of Technology, University of Tartu, Nooruse 1, Tartu 50411, Estonia. [4]Organismal and Evolutionary Biology Research Programme, Faculty of Biological and Environmental Sciences, and Viikki Plant Science Centre, University of Helsinki, Helsinki FI-00014, Finland. [5]Department of Pharmacology, University of California San Diego, La Jolla, CA 92093, USA.
*Corresponding author. Email: ytakahashi@ucsd.edu (Y.T.); jischroeder@ucsd.edu (J.I.S.)
†Present address: Institute of Transformative Bio-Molecules, Nagoya University, Furocho, Chikusa, Nagoya, Aichi 464-8601, Japan.

RESULTS

A previous study reported that the HT1 kinase phosphorylates the CBC1 and CBC2 kinases in vitro (*13*). Whether this phosphorylation affects CBC1/CBC2 kinase activity remains unknown. We confirmed CBC1 and CBC2 phosphorylation by HT1 using recombinant His-HT1 and glutathione *S*-transferase (GST)–CBC1 or GST-CBC2 proteins by in vitro phosphorylation assays using radioactive ^{32}P-ATP (adenosine 5′-triphosphate) (fig. S1). Moreover, phosphorylation levels of histone, an artificial kinase substrate, were increased at the same time. Together with findings that histone is not a substrate of HT1 (e.g., fig. S1), these data suggest that the CBC1 phosphorylation by HT1 may induce CBC1 kinase activation (fig. S1). In-gel kinase assays were pursued to test this hypothesis and provide direct evidence of HT1-induced CBC1 kinase activation (Fig. 1A). In contrast, the kinase inactive HT1-K113W mutant did not activate CBC1 (Fig. 1B). The kinase inactive CBC1-D253A isoform shows a reduced phosphorylation level compared to wild-type (WT) CBC1 and no clear phosphorylation of histone in the presence of HT1 (Fig. 1B). These findings suggest that after CBC1 activation by HT1, the CBC1 protein kinase can mediate autophosphorylation of CBC1 and transphosphorylation of histone (Fig. 1B). We identified in vitro phosphorylation sites in CBC1 using mass spectrometry by analyzing recombinant CBC1 protein in the presence or absence of HT1 (Fig. 1C). We found two HT1-dependent phosphorylation sites (Thr^{256} and Ser^{280}) that lie within or near the activation loop of CBC1. In vitro phosphorylation assays suggest that these two HT1-dependent phosphorylation sites play an important role in HT1-mediated CBC1 activation (Fig. 1D). In contrast, the blue-light–dependent phosphorylation sites (Ser^{43} and Ser^{45}) (*13*) do not have a clear role in HT1-mediated CBC1 activation (Fig. 1D). We created transgenic *Arabidopsis* plants expressing WT CBC1 or CBC1 with amino acid substitutions of the Thr^{256} and Ser^{280} to alanine (T256A/S280A) in the *cbc1 cbc2* double-mutant background under the control of a strong guard cell–expressing promoter, *pGC1* (*15*). Gas exchange experiments revealed that *pGC1:CBC1(T256A/S280A)/cbc1 cbc2* showed a low stomatal conductance and a CO_2-insensitive phenotype, similar to the parent *cbc1 cbc2* double mutant. In contrast, *pGC1:CBC1* rescued the *cbc1 cbc2* phenotype, suggesting that phosphorylation of CBC1 Thr^{256} and Ser^{280} is required for CBC1 function in the stomatal CO_2 response (Fig. 1E).

We tested whether the HT1-mediated activation of CBC1 is inhibited by CO_2/bicarbonate by adding $NaHCO_3$ in in vitro phosphorylation reactions. However, our results show no clear effect of $NaHCO_3$ on the HT1-mediated CBC1 phosphorylation level (Fig. 2A, control lanes 1 and 2; $n > 3$ experiments). Unexpectedly, when MPK4 or MPK12 were added to the reaction, we found that the addition of $NaHCO_3$, but not NaCl, inhibited both CBC1 phosphorylation and histone phosphorylation in vitro (Fig. 2A, MPK4/MPK12 lanes 4 and 6; $n > 6$). However, we did not observe a clear effect of MPK4 or MPK12 without addition of $NaHCO_3$ (Fig. 2A, MPK4/MPK12 lanes 3 and 5). In contrast, the cytosolic domain of the (pseudo-) receptor kinase GUARD CELL HYDROGEN PEROXIDE-RESISTANT1 (GHR1) (*16*, *17*) had no clear effect,

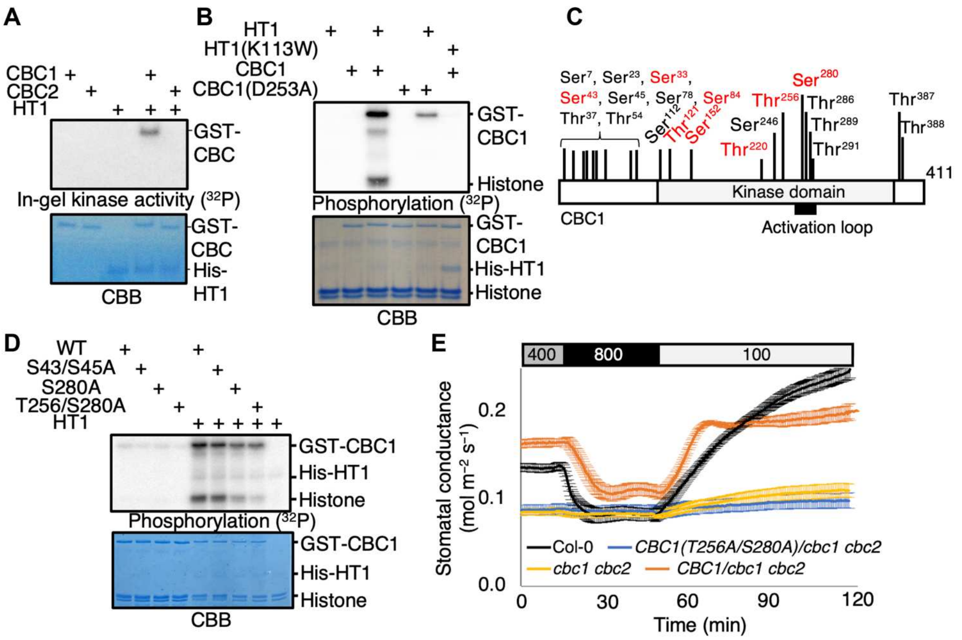

Fig. 1. The CO_2 signaling Raf-like kinase CBC1 is activated by the HT1 protein kinase through phosphorylation. (**A**) Recombinant CBC1 and CBC2 proteins were incubated with or without HT1 proteins for 30 min with ATP, and in-gel kinase assays were performed. (**B**) The kinase inactive CBC1-D253A and HT1-K113W protein isoforms were used for in vitro phosphorylation analyses with recombinant CBC1 and HT1 proteins as indicated (see main text). Histone was used as an artificial phosphorylation substrate of CBC1. (**C**) Recombinant CBC1 proteins were incubated with or without HT1 and ATP, and CBC1 phosphorylation sites were identified by mass spectrometry. The red fonts indicate HT1-dependent in vitro phosphorylation sites. CBC1 autophosphorylation sites detected without HT1 addition are labeled in black fonts. The Ser^{43} and Ser^{45} were previously reported as blue light–dependent phosphorylation sites (*13*) but, when mutated alone, did not affect HT1 activation of CBC1 (D). (**D**) CBC1-S43/S45A, S280A, and T256/S280A proteins were used for in vitro phosphorylation assays. CBB gels show loading controls. (**E**) Stomatal conductances were analyzed using intact leaves attached to intact *Arabidopsis* plants (Col-0, *cbc1 cbc2*, *pGC1:CBC1-GFP/cbc1 cbc2*, and *pGC1:CBC1(T256A/S280A)-GFP/cbc1 cbc2*). CO_2 concentration changes were applied as indicated on top (parts per million).

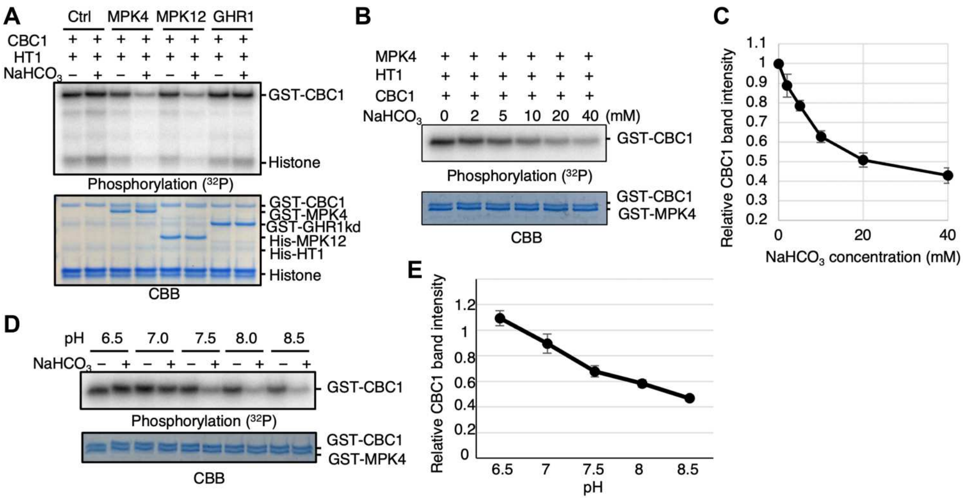

Fig. 2. MAP kinases MPK4 and MPK12 inhibit HT1-mediated CBC1 kinase phosphorylation in the presence of elevated $NaHCO_3$ in vitro. (**A**) Recombinant HT1 and CBC1 proteins were incubated with MPK4, MPK12, or the (pseudo)-kinase domain of GHR1 in the presence or absence of 20 mM $NaHCO_3$ for 30 min, and in vitro phosphorylation assays were performed. Histone was used as an artificial protein kinase substrate. (**B**) MPK4, HT1, and CBC1 proteins were incubated with $NaHCO_3$ at the indicated concentrations for 30 min, and in vitro phosphorylation assays were performed. (**C**) CBC1 band intensities as shown in (B) were measured using ImageJ. $n = 4$ experiments. Error bars show ±SD. (**D**) MPK4, HT1, and CBC1 proteins were incubated in reaction buffers adjusted at different pH (6.5 to 8.5) for 30 min, and in vitro phosphorylation assays were performed. (**E**) CBC1 band intensities and the density ratios of "+$NaHCO_3$" to "−$NaHCO_3$" (= +NaCl controls) for each pH condition as shown in (D) were measured using ImageJ. $n = 4$ experiments. Error bars show ±SD.

further indicating a function of MPK4 and MPK12 (Fig. 2A, GHR1 lanes).

The inhibitory down-regulation of CBC1 activity shows a $NaHCO_3$ dose dependency (Fig. 2, B and C). The EC_{50} (median effective concentration) of the inhibited activity was ~7.1 ± 1.0 mM in vitro under the imposed conditions and protein concentrations, which is similar to the unrelated cyanobacterium adenylyl cyclase bicarbonate sensor (*18*) and the mammalian soluble adenylyl cyclase bicarbonate sensor (*19*). In control "-$NaHCO_3$" experiments, we used the same concentration of NaCl, which did not cause CBC1 activity regulation (Fig. 2). When in vitro phosphorylation assays were performed using reaction buffers adjusted to different pH values individually, $NaHCO_3$ inhibited CBC1 phosphorylation in the presence of MPK4 and HT1 under high pH conditions at ≥pH 7.5 (Fig. 2, D and E), which suggests that bicarbonate ions are the main inorganic carbon signaling species. We note that these results do not necessarily exclude a secondary role of CO_2, which is more abundant at low pH, although bicarbonate ions clearly have the stronger effect on kinase regulation (Fig. 2, D and E). A human soluble adenylyl cyclase, for example, senses both CO_2 and bicarbonate ions (*20*).

We found that MPK11, a MPK member from the same *Arabidopsis* MPK subfamily as MPK4 and MPK12, did not mediate $NaHCO_3$-induced inhibition of CBC1 phosphorylation in in vitro phosphorylation assays (fig. S2A; $n > 5$), whereas MPK12 inhibited CBC1 phosphorylation in the presence of $NaHCO_3$ (fig. S2A), which is consistent with previous studies suggesting that MPK11 does not contribute measurably to CO_2 signaling in guard cells (*14*, *21*). Furthermore, we tested MPK3 from another *Arabidopsis* MPK family, which has diverse roles in plant stress signal transduction pathways redundantly with MPK6. We found that MPK3 had no role in inhibition of CBC1 phosphorylation unlike MPK4 and MPK12 (fig. S2B; $n > 5$).

In parallel to these analyses, a genetic screen was pursued for ozone-sensitive *Arabidopsis* mutants, which can result from higher stomatal conductance mutant phenotypes that enable damaging access of ozone into intercellular leaf spaces (*17*, *22*). Screening of >50,000 ethylmethane sulfonate–mutagenized M2 generation *Arabidopsis* lines led to isolation of candidate mutants impaired in the stomatal high CO_2 response while exhibiting intact abscisic acid–induced stomatal closing (see Materials and Methods). These mutants included six mutants in the *HT1* gene, comprising *ht1-G89R* and *ht1-R173Q* alleles and reisolations of the known *ht1-A109V* [*ht1-8D* in (*23*)] variant in four remaining mutants. All of these *ht1* mutant alleles were dominant and showed stomatal insensitivity to CO_2 elevation (Fig 3, A, B, and F). Whole-plant gas exchange analyses revealed that the *ht1-G89R* mutant showed an increased stomatal conductance at ambient CO_2 that did not respond to changes in the CO_2 concentration (Fig. 3A and fig. S3A). The stomatal conductance of the *ht1-G89R* mutant was smaller than that of WT control plants at low [CO_2] [100 parts per million (ppm) CO_2; Fig. 3A]. The *ht1-R173Q* mutant showed partly impaired stomatal conductance responses to CO_2 shifts (Fig. 3B).

Both recombinant HT1-G89R and HT1-R173Q proteins activated CBC1 protein in vitro (Fig. 3C, lanes 1, 2, 4, and 6). However, the HT1-G89R protein activated CBC1 protein less than WT HT1 protein (Fig. 3C, lane 2 versus lane 4). In vitro phosphorylation assays revealed that the HT1-G89R isoform shows no $NaHCO_3$-mediated inhibition of CBC1 activity, in contrast to the WT HT1 protein (Fig. 3C; $n = 4$ experiments). Furthermore, the R173Q mutation partly impaired the $NaHCO_3$-dependent CBC1 down-regulation (Fig. 3C). These results are consistent with the stomatal phenotypes of the mutant plants (Fig. 3, A to C). The smaller stomatal conductance of the *ht1-G89R* mutant plants than that of WT plants in response to low CO_2 conditions (100 ppm CO_2;Fig. 3A) is

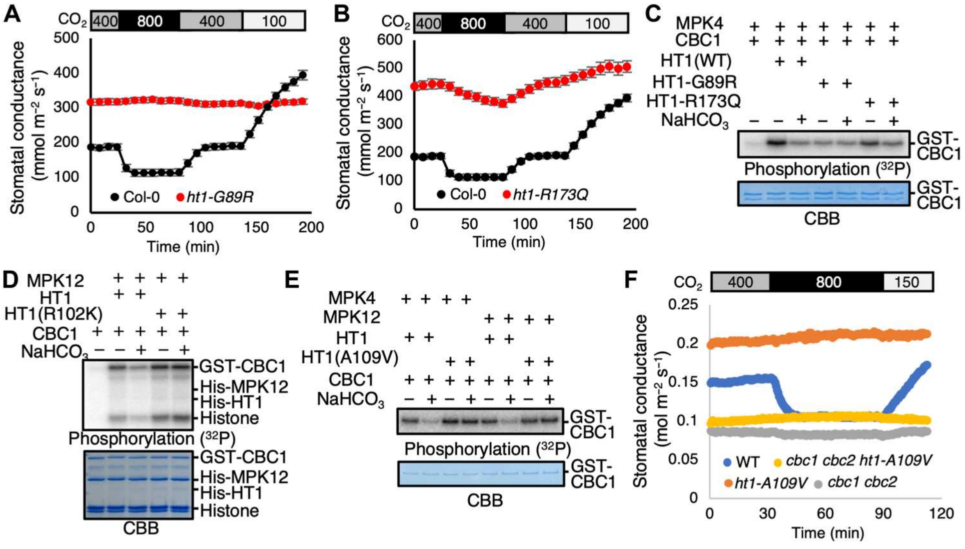

Fig. 3. The dominant HT1 mutations (HT1-R102K and A109V) disrupt HCO_3^--dependent down-regulation of CBC1 protein kinase activity. (**A** and **B**) Whole-plant gas exchange analyses using *ht1-G89R* (A) and *ht1-R173Q* (B). Ambient CO_2 concentrations are indicated by the top bars. $n = 7$ experiments. Error bars show ± SEM. (**C**) Recombinant HT1 (WT, HT1-G89R, or HT1-R173Q) and CBC1 proteins were incubated with MPK4 in the presence or absence of 20 mM $NaHCO_3$ or 20 mM NaCl ("−" controls) for 30 min, and in vitro phosphorylation assays were performed. (**D**) Recombinant MPK12, CBC1, and HT1 (WT or R102K) proteins were incubated with or without 20 mM $NaHCO_3$ or 20 mM NaCl (− controls) for 30 min, and in vitro phosphorylation assays were performed. Histone was used as an artificial kinase substrate. (**E**) Recombinant HT1 (WT or A109V) proteins were used for in vitro phosphorylation assays with MPK4 or MPK12 and CBC1 proteins. Proteins were incubated with or without 20 mM $NaHCO_3$ or 20 mM NaCl (− controls) for 30 min. (**F**) Stomatal conductances were analyzed using intact plants of *Arabidopsis* [Col-0 (WT), *cbc1 cbc2*, *ht1-A109V* , and *cbc1 cbc2 ht1-A109V*]. CO_2 concentration changes were applied as indicated on top (parts per million).

consistent with the lower kinase activity of the HT1-G89R isoform at low CO_2/bicarbonate concentrations (Fig. 3C, lane 2 versus lane 4). Whole-plant gas exchange analyses showed that the more strongly dominant CO_2-insensitive *ht1-A109V* mutant plants (*23*) showed a greater stomatal conductance than the *ht1-G89R* mutant plants (fig. S3A). These CO_2-insensitive mutants had no obvious effect on photosynthesis-mediated CO_2 uptake under the imposed conditions (fig. S3B). We examined additional dominant *ht1* mutants. In contrast to recessive *ht1* kinase mutants (*12*), two strong dominant *ht1* mutations, *ht1-R102K* [*ht1-3* in (*24*)] and *ht1-A109V*, cause a constitutively open and high CO_2-insensitive stomata phenotype (*23*, *24*). These data suggest that these dominant mutations constitutively enhance HT1 function in guard cells. However, these mutations do not greatly enhance HT1 kinase activity (*23*, *24*). In our phosphorylation assays using MPK4/12, HT1, and CBC1 recombinant proteins, both of these R102K and A109V HT1 mutations disrupt the $NaHCO_3$-triggered down-regulation of CBC1 phosphorylation and CBC1 activity [Fig. 3, D and E; $n = 3$ (D) and $n = 4$ (E) experiments].

The above results suggest that our in vitro signaling analyses can explain how these HT1 point mutations confer their CO_2-insensitive stomatal phenotypes. In a model derived from the above findings, low CO_2-induced activation of the CBC1 kinase requires CBC1 phosphorylation by HT1. CBC1 activity, in turn, is down-regulated by high CO_2/bicarbonate down-regulation of HT1. A prediction of this model would be that the constitutively open stomatal phenotypes of the dominant *ht1-A109V* mutant would require the presence of the CBC kinases. We created *cbc1 cbc2 ht1-A109V* triple mutant plants. Stomatal conductance analyses show that the triple mutant has a closed stomatal phenotype, somewhat similar to *cbc1 cbc2* mutant leaves, whereas *ht1-A109V* single-mutant leaves have constitutively open stomata (Fig. 3F). This genetic evidence supports the model that HT1 provides a critical upstream regulator connecting CO_2 sensing to downstream CBC kinase activity regulation.

Additional experiments including a strong recessive *ht1-2* mutant (*12*) revealed that the *cbc1 cbc2* double mutant and *cbc1 cbc2 ht1-A109V* triple mutant have a slightly greater stomatal conductance when compared to the *ht1-2* mutant, whose stomatal conductance is consistently very low (fig. S4). *cbc1 cbc2* double-mutant leaves showed a very weak CO_2 response (fig. S4). These results may suggest that an additional member(s) of the three CBC homologous proteins from the C7 subgroup of the Raf-like kinase family (*13*) have an overlapping (genetically redundant) function together with CBC1 and CBC2 in mediating stomatal opening in response to low CO_2 conditions.

We further pursued experiments to identify the molecular mechanism of CO_2/bicarbonate sensing. When CBC1, HT1 or MPK4 were exposed to high $NaHCO_3$ individually, their kinase activities were not affected (Fig. 4A; $n > 3$), in the case of the MPKs, consistent with previous findings showing no direct MPK4 and MPK12 activation by elevated CO_2 or $NaHCO_3$ (*14*). However, HT1 kinase activity was inhibited in response to $NaHCO_3$ in the presence of either MPK4 or MPK12 (Fig. 4B; $n > 3$; fig. S5). In contrast, CBC1 activity was not affected when MPK4 or MPK12 and CBC1 were added to the reaction without HT1 protein (Fig. 4B; $n > 3$; fig. S5), suggesting that MPK4, MPK12, and HT1 might be the bicarbonate sensing module.

We therefore investigated possible binding between MPKs and HT1 and the effect of HCO_3^-. In vitro pull-down assays showed that HCO_3^- greatly enhanced the binding between MPK4 and HT1 (Fig. 4C; $n > 6$ experiments). Similar experiments revealed

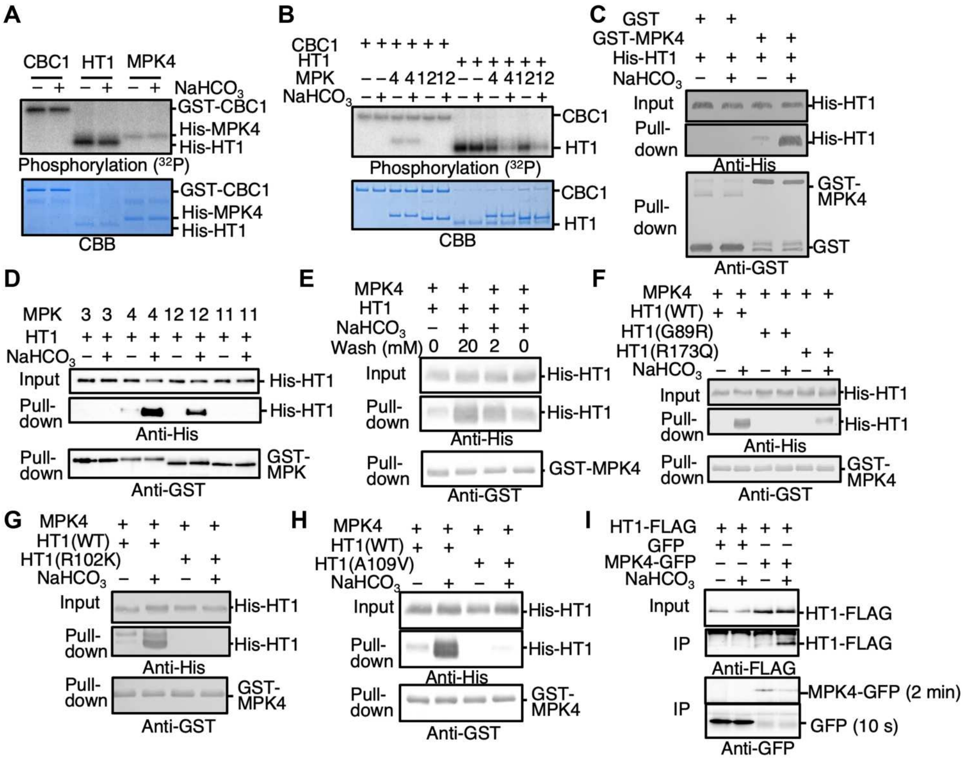

Fig. 4. Bicarbonate inactivates HT1 kinase by stabilizing HT1 interaction with MPK4/12. (**A**) Recombinant CBC1, HT1, and MPK4 proteins were incubated with or without 20 mM $NaHCO_3$ for 30 min, and in vitro phosphorylation assays were performed. (**B**) CBC1 and HT1 proteins were incubated with or without 20 mM $NaHCO_3$ or 20 mM NaCl (− controls) in the presence or absence of MPK4 or MPK12 protein. (**C**) His-HT1 and GST-MPK4 or GST control proteins were used for in vitro pull-down assays with or without 20 mM $NaHCO_3$. $NaHCO_3$ or 20 mM NaCl (− controls) were supplemented in all buffers throughout the pull-down assay procedures including the washing step. (**D**) In vitro pull-down assays were performed using His-HT1 and GST-MPK3, GST-MPK4, GST-MPK12, or GST-MPK11 proteins. (**E**) In vitro pull-down assays showed reversibility and were performed using the washing buffers supplemented with $NaHCO_3$ at the indicated concentrations (0, 2, or 20 mM). (**F**) In vitro pull-down assays were performed using recombinant HT1 (WT, HT1-G89R, and HT1-R173Q) proteins. (**G**) In vitro pull-down assays were performed using HT1-R102K isoform. (**H**) In vitro pull-down assays were performed using HT1-A109V isoform. (**I**) HT1-FLAG and MPK4-GFP or GFP (control) were transiently expressed in *Arabidopsis* mesophyll cell protoplasts. Coimmunoprecipitation analyses using polyclonal GFP antibodies were performed, and then, precipitated proteins were detected by immunoblot analyses using monoclonal FLAG or GFP antibodies. The immunoblot images showing MPK4-GFP and GFP bands were from the same single membrane, but exposure times are different as indicated next to the images (2 min for MPK4-GFP and 10 s for GFP control) because the MPK4-GFP expression levels were much lower than those of the GFP control. IP, immunoprecipitation.

that MPK12 also interacted with HT1, but other MPKs, MPK3 and MPK11, showed no binding to HT1 protein (Fig. 4D). This HCO_3^--dependent binding was reversed by removing HCO_3^-, indicating that MPK4 and HT1 interact reversibly depending on the bicarbonate concentration (Fig. 4E and fig. S6). The HT1-G89R mutation disrupts the HCO_3^--dependent interaction of HT1 with MPK4 (Fig. 4F; $n = 3$ experiments). The HT1-R173Q isoform, which causes a weaker CO_2-insensitive phenotype (Fig. 3B), is still able to partially interact with MPK4 upon HCO_3^- addition, albeit less strongly than the WT MPK4-HT1 proteins (Fig. 4F; $n = 3$ experiments). Furthermore, the strong dominant HT1-R102K and HT1-A109V mutant isoforms did not show a bicarbonate-induced interaction of HT1 with MPK4 (Fig. 4, G and H; $n = 3$ experiments each). These findings are consistent with in vitro phosphorylation assays (Fig. 3, C to E) and stomatal conductance analyses (Fig. 3, A, B, and F). Furthermore, blinded quantitative bimolecular fluorescence complementation (BiFC) analyses showed that CO_2 elevation enhanced interactions between MPK4/MPK12 and HT1 in plant cells, whereas a control interaction between ABA-INSENSITIVE1 (ABI1) and guanine nucleotide exchange factor 1 (GEF1) (*25*) occurred constitutively independent of the CO_2 concentration, suggesting that MPK4 and MPK12 interact with HT1 in response to CO_2 elevation in plant cells (Fig. 5). In addition, coimmunoprecipitation analyses provide evidence that HCO_3^- induces an interaction between MPK4 and HT1 proteins transiently expressed in *Arabidopsis* mesophyll cell protoplasts (Fig. 4I and fig. S7). Together, these results suggest that MPK4/MPK12 and HT1 are the long-sought CO_2/bicarbonate sensor, for which HCO_3^- causes an interaction of MPK4/12 with HT1, which in turn inhibits the negative regulatory HT1 protein kinase activity, thus enabling high CO_2-induced stomatal closure to proceed.

Initial AlphaFold2 modeling and Gaussian-accelerated molecular dynamics (GaMD) simulations (*26*–*30*) of binary complexes of MPK4 and MPK12 with HT1 predict that the dominant HT1 mutant residues, A109V, R102K, and G89R, notably cluster at the interface of HT1 with MPK4 and MPK12 (Fig. 6, A and B). These dominant mutations are predicted to reduce the HT1-MPK12 binding affinity, thus destabilizing the interaction of HT1 with MPK12 (table S1) and may therefore provide an explanation for the impairment in the bicarbonate-induced interaction of these

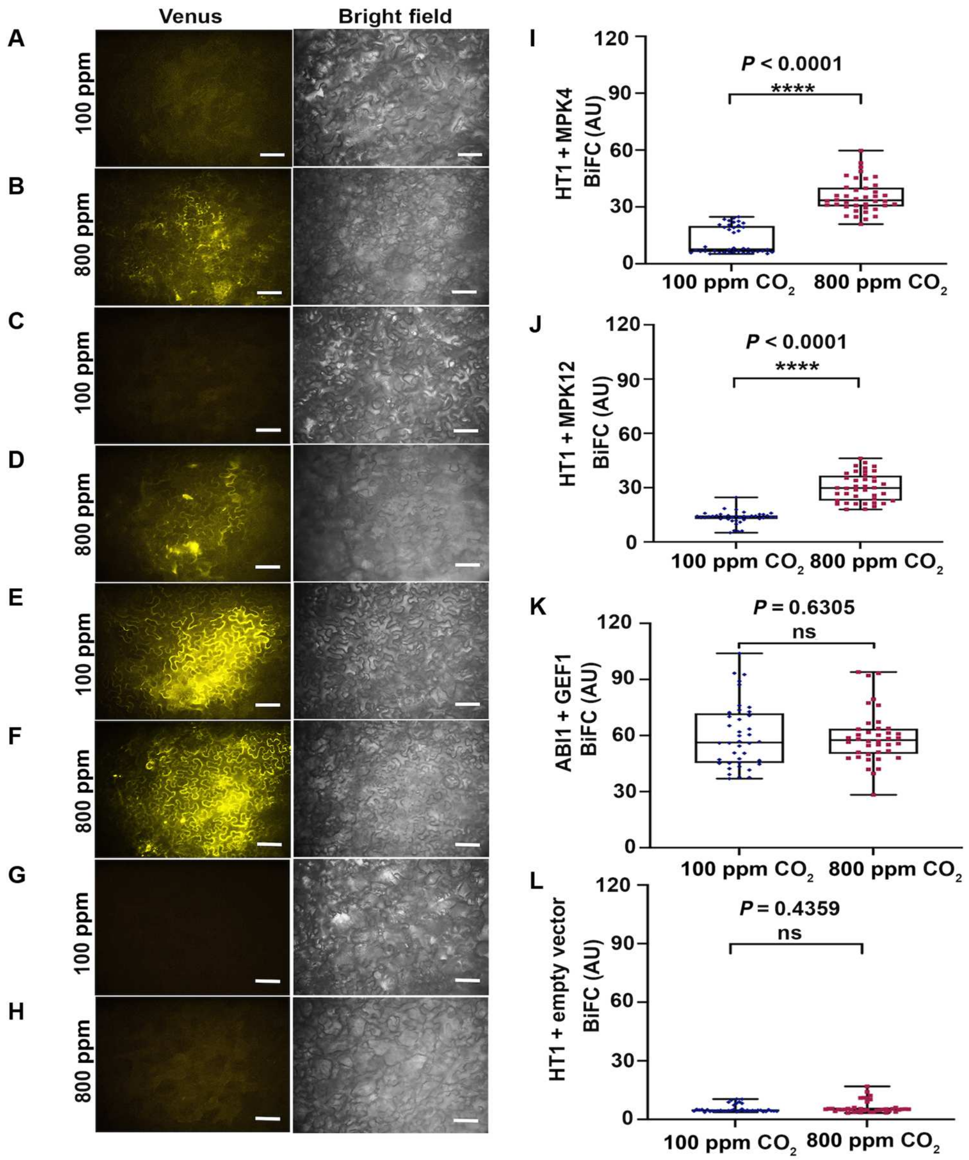

Fig. 5. Interaction of MPK4 and MPK12 with HT1 in response to CO_2 increase. Inoculant-blinded BiFC experiments with split-Venus fragments fused to HT1 and MPK4 (**A**) at nominally 100 ppm CO_2 and (**B**) at nominally 800 ppm CO_2, HT1 and MPK12 (**C**) at 100 ppm CO_2 and (**D**) at 800 ppm CO_2, ABI1 and GEF1 (positive control) (**E**) at 100 ppm CO_2 and (**F**) at 800 ppm CO_2, HT1 and empty vector (negative control) (**G**) at 100 ppm CO_2 and (**H**) at 800 ppm CO_2 expressed in *N. benthamiana* leaves. Representative BiFC images with brightness contrast being identical in all images and identical confocal settings are shown. Scale bars, 20 μm. (**I** to **L**) Boxplot analysis of inoculant-blinded BiFC image intensities indicates enhanced interactions of (I) MPK4 with HT1 and (J) MPK12 with HT1 when plants were exposed to 800 ppm ambient CO_2 compared to 100 ppm CO_2. In contrast, positive control interaction assays (K) using ABI1 and GEF1 (*25*) and negative control (L) HT1 and empty vector showed no significant difference at both CO_2 concentrations. Inoculants were blinded to the experimenter and unblinded by an independent person after the experimentor had analyzed all images and completed blinded data sets and beeswarm boxplots. Statistical analyses of the BiFC data were performed using Mann-Whitney test [ns (not significant), $P > 0.05$; ****$P < 0.0001$]. Normality of variable was evaluated by the D'Agostino-Pearson omnibus normality test. The values were plotted in beeswarm box and whiskers (range minimum to maximum) plots. Ten images were analyzed for each condition, and four nonoverlapping areas were analyzed and then averaged for each image. For statistical analyses, "*n*" is equal to number of images. All graphs and statistical analyses were performed using GraphPad Prism Software (version 9.0.0). AU, arbitrary units.

proteins and impairment in the CO_2/HCO_3^--induced inhibition of HT1 protein kinase activity found for these dominant mutants. The R173 residue that caused a distinct and weaker phenotype in the *ht1-R173Q* mutant is predicted to lie proximal to the MPK4/12-HT1 interface but not within the A109/R102/G89 cluster (Fig. 6, A and B). In addition to the above forward genetically isolated dominant HT1 mutations, using this structural model and simulations (see Materials and Methods), possible mutations that may impair the interaction of MPK12 with HT1 were computationally derived. The amino acid mutation MPK12-Y277G was predicted to have a strong impact on reducing the binding affinity of MPK12 to HT1 (table S2). In vitro pull-down experiments

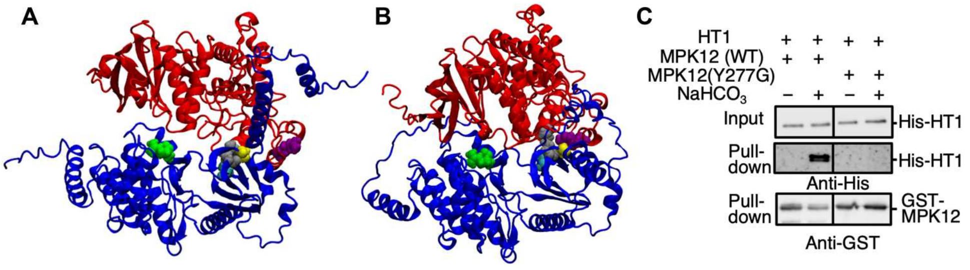

Fig. 6. AlphaFold2-predicted complexes of MPK4-HT1 and MPK12-HT1. (**A** and **B**) Predicted complexes of MPK4-HT1 (A) and MPK12-HT1 (B). MPK4 (A) and MPK12 (B) are shown in red, while HT1 is in blue. The residues are colored as follows: G89-HT1 (yellow), R102-HT1 (silver/gray), A109-HT1 (cyan), R173-HT1 (lime green), and Y277-MPK4/12 (purple). G89, R102, and A109 are predicted to form a cluster. MPK12-Y277 is proximal to the dominant mutation cluster in HT1 (A109/R102/G89) in the MPK12-HT1 complex (B) but slightly more distant in the MPK4-HT1 complex (A). (**C**) In vitro pull-down assays were performed using HT1 and MPK12 and the MPK12-Y277G isoform.

showed disruption of the CO_2/HCO_3^--induced interaction of MPK12-HT1 (Fig. 6C). In planta BiFC experiments further showed impairment in the ability of elevated CO_2 to enhance MPK12-Y277G interaction with HT1 (fig. S8).

Unexpectedly, we found that the kinase inactive MPK12-K70R isoform (*21*) retained the ability to mediate CO_2/bicarbonate-induced CBC1 inhibition via the HT1 protein kinase in in vitro phosphorylation assays (Fig. 7A, $n = 4$). The kinase inactive MPK4-K72M/K73R (*23*) was also able to mediate CO_2/bicarbonate-induced CBC1 inhibition in vitro (fig. S9). We further investigated the requirement of MPK activity for CO_2 regulation of stomatal movements in planta. Strong *mpk12* mutant alleles show a larger steady state stomatal conductance and slightly slowed high CO_2 responses (*14*, *21*, *23*). Consistent with phosphorylation analyses (Fig. 7A), the inactive MPK12-K70R isoform rescued the open

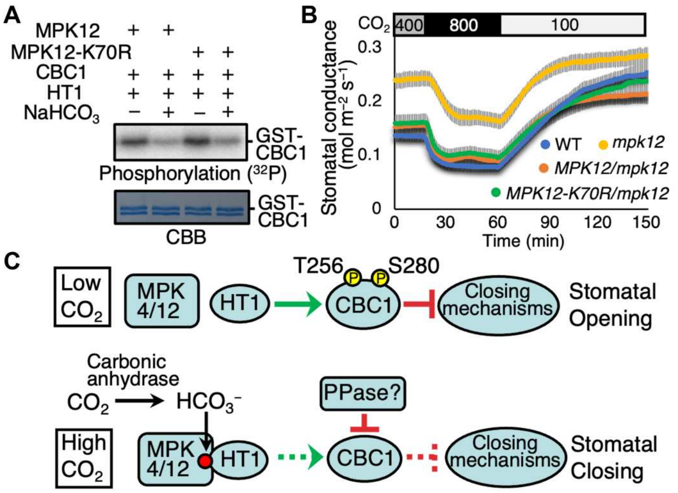

Fig. 7. The kinase inactive MPK12 isoform is sufficient for stomatal CO_2 sensing. (**A**) In vitro phosphorylation assays were performed after CBC1, HT1 and MPK12, or the MPK12-K70R kinase inactive isoform proteins were incubated with or without $NaHCO_3$ or NaCl (−) controls. (**B**) Stomatal conductances were analyzed in leaves of intact *Arabidopsis* plants [Col-0 (WT), *mpk12*, *pGC1:MPK12-GFP/mpk12* and *pGC1:MPK12(K70R)-GFP/mpk12*]. CO_2 concentration changes were applied as indicated on top (parts per million). $n = 6$ experiments. Error bars show ± SEM. (**C**) Model of plant stomatal CO_2 sensor and signaling (see main text). An unknown protein phosphatase (PPase) is assumed to inhibit CBC1 at high CO_2 for activation of stomatal closing mechanisms.

and slowed stomatal CO_2 response phenotype of *mpk12* mutant leaves (Fig. 7B). Complementation of the in planta *mpk12* CO_2 response was similar upon expression of the kinase dead MPK12-K70R kinase or the WT MPK12 isoforms. These findings further suggest that the signaling mechanism by which the HT1 and MPK12 protein kinases sense CO_2 concentration functions via a reversible MPK-HT1 interaction rather than HT1 phosphorylation by these MAP kinases.

DISCUSSION

In this study, we reveal the biochemical, genetic, and physiological stomatal CO_2 sensing and early signaling core mechanisms that use three types of protein kinases, MPK4/MPK12, HT1, and CBC1. While HCO_3^- can modulate ~20% of the activity of downstream S-type anion channels, as a secondary HCO_3^- sensing mechanism (*10*), the primary CO_2/bicarbonate sensors that control the required upstream phosphorylation events and thereby stomatal closing have remained elusive. The present findings that the MPK4/MPK12-HT1 complex functions as a bicarbonate sensor together with strong genetic CO_2-insensitive phenotypes of the respective *ht1* and *cbc1 cbc2* mutants (Fig. 3, A, B, and F) (*12*, *13*) provides a model for how plant cells sense and transmit the CO_2 signal to trigger stomatal closure. At low CO_2/bicarbonate concentrations, the HT1 kinase phosphorylates and activates the CBC1 protein kinase, which leads to inhibition of stomatal closing mechanisms (Fig. 7C). However, when guard cells are exposed to high CO_2 concentrations, carbonic anhydrases accelerate the intracellular conversion of CO_2 to bicarbonate (*7*, *8*), and the accumulated bicarbonate ions can trigger MPK4/12-HT1 binding that leads to inhibition of HT1 kinase activity. HT1 kinase inhibition in turn results in down-regulation of CBC1 kinase activity promoting induction of stomatal closure (Fig. 7C). The kinase inactive MPK12 isoform is sufficient for stomatal CO_2 sensing and the in planta complementation of the stomatal CO_2 response (Fig. 7, A and B), suggesting an unexpected phosphorylation-independent MAP kinase function in plants. The reversible MPK4/MPK12-HT1 binding (Fig. 4E and fig. S6) further correlates with the rapid reversibility of stomatal opening and closing in response to changing CO_2 concentrations in leaves (*1*, *3*).

Our signaling model can further explain the phenotypes of previously isolated dominant CO_2-insensitive mutations of *HT1*. Although both HT1-R102K and HT1-A109V kinases have a similar kinase activity to WT HT1 kinase, CO_2 elevation cannot induce a

strong interaction between these HT1 isoforms and MPK4 and MPK12. This failure of interaction keeps these HT1 isoforms active even at elevated CO_2, resulting in continuous CBC1 kinase activity (Fig. 3, D and E), which causes a constitutive open stomatal phenotype (*23*, *24*). The previously unidentified *HT1-G89R* and *HT1-R173Q* mutations may have a similar, but not identical strengths of their effect, in that the HT1-R173Q effect appears to be partial and the HT1-G89R may affect kinase activity itself (Figs. 3, A to C and 4F).

Physiological bicarbonate concentrations in guard cells have not been clearly determined to date and could be lower than the concentrations used in in vitro analyses. An additional mechanism such as a HCO_3^- concentrating mechanism, unknown scaffold, other factors, local compartmentation mechanisms, and/or posttranslational modifications of MPK4/12 or HT1 may be active in planta and need to be investigated in future research. Initial AlphaFold2-directed modeling combined with molecular dynamics simulations predict that the dominant HT1 mutant residues, A109V, R102K, and G89R, cluster at the interface of HT1 with MPK4 and HT1 with MPK12 (Fig. 6, A and B). Moreover, the computationally predicted MPK12-Y277G variant (table S2) that impairs bicarbonate-induced MPK12-HT1 interaction (Fig. 6C and fig. S8) lies in close proximity to this HT1-A109/R102/G89 cluster (Fig. 6B). Further structural resolution, molecular dynamics simulations and site-directed mutagenesis can test this model. Research is needed to elucidate the structure and binding of CO_2/bicarbonate to the identified HT1-MPK4/MPK12 complex.

Previous studies suggest that natural variation in *MPK12* is linked to water use efficiency differences in *Arabidopsis* ecotypes (*21*, *31*, *32*). The identification of the guard cell HT1-MPK4/MPK12 CO_2 sensor and the mechanisms within the MPK4/12-HT1-CBC1 and CBC2 CO_2 signaling core that regulate stomatal conductance in the present study could lead to future targeted engineering of plant water use efficiency and carbon intake in light of the continuing increase in the atmospheric CO_2 concentration (*2*, *6*, *33*, *34*).

MATERIALS AND METHODS

Vector constructions

For the expression of GST-tagged and His-tagged proteins, pGEX-6P-1 and pET30a(+) *Escherichia coli* expression vectors were used, respectively. Primer sequences used for cloning in this study are provided in table S1.

Producing recombinant proteins

Recombinant proteins were produced using an *E. coli* protein expression system. Briefly, bacteria were grown in LB or 2xYT medium at 37°C until the optical density at 600 nm (OD_{600}) reached ~0.5 to 0.7, then 0.5 mM isopropyl-β-d-thiogalactopyranoside was added, and *E. coli* were incubated at 20°C for 16 to 24 hours. *E.coli* cells were harvested by centrifugation at 2000*g* for 20 min and resuspended in tris-buffered saline [50 mM tris-HCl (pH 7.5) and 150 mM NaCl]. The *E. coli* cells were disrupted by ultrasonication, and extracted proteins were separated from cell debris by centrifugation at 14,000*g* for 10 min. GST-tagged proteins and His-tagged proteins were purified using glutathione Sepharose beads and Ni resin beads, respectively.

Phosphorylation assays

GST-CBC1, GST- or His-MPK4, His-MPK12, His-MPK3, His-MPK11, His-GHR1, and His-HT1 proteins were produced using *E. coli* expression. Proteins were incubated in 20 μl of phosphorylation buffer [50 mM tris-HCl (pH 7.5), 10 mM $MgCl_2$, 0.1% Triton X-100, and 1 mM dithiothreitol (DTT)] with 200 μM ATP and 1 μCi [γ-^{32}P]-ATP for 30 min at room temperature. $NaHCO_3$ and NaCl, as controls, were added 30 min before to protein reaction solutions. Reactions were stopped by the addition of SDS–polyacrylamide gel electrophoresis (SDS-PAGE) loading buffer. Exact protein amounts used in each experiment were as follows: 0.8 μg of GST-CBC1 and 0.8 μg of His-HT1 (Fig. 1B); 1 μg of GST-CBC1 and 0.5 μg of His-HT1 (Fig. 1D); 0.6 μg of GST-CBC1, 0.3 μg of His-HT1, and 1 μg of GST-MPK4, His-MPK12, or GST-GHR1 (Fig. 2A); 0.5 μg of GST-CBC1, 0.5 μg of GST-MPK4, and 0.01 μg of His-HT1 (Fig. 2, B and D); 0.5 μg of GST-CBC1, 0.5 μg of GST-MPK4, and 0.01 μg of His-HT1 (WT, G89R, or R173Q) (Fig. 3C); 0.5 μg of GST-CBC1, 1 μg of His-MPK12, and 0.02 μg of His-HT1 (Fig. 3D); 0.5 μg of GST-CBC1, 0.5 μg of His-MPK4 or His-MPK12, and 0.01 μg of His-HT1 (Fig. 3E); 1 μg of GST-CBC1, 0.4 μg of His-HT1, and 0.6 μg of His-MPK4 (Fig. 4A); 1 μg of His-MPK4 or His-MPK12 and 1 μg of GST-CBC1 or 0.5 μg of His-HT1 (Fig. 4B); 0.5 μg of GST-CBC1, 0.5 μg of GST-MPK12 (WT or K70R), and 0.01 μg of His-HT1 (Fig. 7A); 0.4 μg of GST-CBC1 and 0.4 μg of His-HT1 (fig. S1); 0.5 μg of GST-CBC1, 0.5 μg of GST-MPK3, MPK4, MPK11, or MPK12; and 0.01 μg of His-HT1 (fig. S2); and 0.5 μg of GST-CBC1, 0.4 μg of His-MPK12, and 0.02 μg His-HT1 (fig. S9). Radiography SDS-PAGE gels were exposed for 1 to 15 hours depending on the band intensity and whether the basal activity of the CBC1 kinase was investigated. For Fig. 2D, the pH of phosphorylation buffers was adjusted using 50 mM MOPS-NaOH (for pH 6.5 and 7.0 buffers) or 50 mM tris-HCl (for pH 7.5, 8.0, and 8.5 buffers). Under the imposed conditions, 20 mM $NaHCO_3$ causes a small pH increase at low pH conditions (0.3 and 0.1 unit in the pH 6.5 and 7.0 buffers, respectively) but only almost negligible changes in higher pH buffers (up to 0.03 pH unit in the pH 7.5 buffer). To ensure low CO_2 concentrations, the phosphorylation buffers and water used for phosphorylation assays were stored in a plant growth chamber adjusted at ambient imposed 100 to 150 ppm CO_2 or to nominally <100 ppm CO_2 by storing buffers in an airtight closed container filled with sodalime.

In-gel kinase assays

Proteins were solubilized in SDS-PAGE loading buffer and separated in acrylamide gels containing casein (0.5 mg/mL). In-gel kinase assays were performed as described previously (*35*). Briefly, gels were incubated in 30 ml of washing buffer [25 mM tris-HCl (pH 8.0), 0.5 mM DTT, 0.1 mM Na_3VO_4, 5 mM NaF, bovine serum albumin (0.5 mg/ml), and 0.1% Triton X-100] for 30 min three times and in 30 ml of renaturation buffer [25 mM tris-HCl (pH 8.0), 1 mM DTT, 0.1 mM Na_3VO_4, and 5 mM NaF] for 30 min once. Gels were further incubated in 30 ml of renaturation buffer at 4°C overnight, followed by further incubation in 20 ml of reaction buffer [50 mM tris-HCl (pH 7.5), 10 mM $MgCl_2$, 2 mM DTT, and 1 mM EGTA] for 30 min. Phosphorylation reactions were carried out in reaction buffer with 50 μCi [γ-^{32}P]-ATP for 60 min at room temperature. Gels were washed in 40 ml of 5% trichloroacetic acid and 1% phosphoric acid four times for 30 min each. Storage phosphor

screen (BAS-IP MS 2025, Fujifilm Corporation, Tokyo, Japan) was used for detection.

In vitro pull-down assays

Five micrograms of His-HT1 and GST-MPK4, GST-MPK12, GST-MPK3, GST-MPK11, or GST control proteins were incubated in 200 μl of buffer [50 mM tris-HCl (pH 7.5), 150 mM NaCl, 0.1% Triton X-100, and 1 mM DTT] with 20 mM $NaHCO_3$ or 20 mM NaCl (controls) for 15 min at room temperature. Ten microliters from solutions from each protein solution were transferred to new tubes as "input" samples. Then, the protein solutions were incubated with 10 μl of glutathione Sepharose 4B beads for 30 min at room temperature. The beads were washed with 1 ml of T-TBS [50 mM tris-HCl (pH 7.5), 150 mM NaCl, and 0.05% Tween-20] supplemented with 20 mM $NaHCO_3$ or 20 mM NaCl three times, and proteins on the glutathione Sepharose beads were solubilized in 25 μl of SDS-PAGE loading buffer. Proteins were detected by immunoblot analyses using anti-GST or anti-His antibodies. To ensure low CO_2 concentrations, the phosphorylation buffers and water used for in vitro pull-down assays were stored in a plant growth chamber adjusted at 100 to 150 ppm CO_2 or at nominally <100 ppm CO_2 by storing buffers in an airtight closed container filled with sodalime.

Phosphorylation site mapping using mass spectrometry

Ten micrograms of GST-CBC1 protein was incubated with or without 10 μg of His-HT1 in 200 μl of phosphorylation buffer [50 mM tris-HCl (pH 7.5), 10 mM $MgCl_2$, 0.1% Triton X-100, and 1 mM DTT] with 1 mM ATP for 1 hour at room temperature. Proteins were precipitated by acetone precipitation and dissolved in SDS-PAGE loading buffer. After SDS-PAGE and Coomassie Brilliant Blue (CBB) staining, protein bands of GST-CBC1 were excised and analyzed by liquid chromatography–tandem mass spectrometry (*35*).

Stomatal conductance analyses

For Figs. 1E, 3F, and 7B, plants grown for gas exchange experiments were grown in potting soil and placed into a plant growth chamber (AR-41L2, Percival Scientific, Perry, IA, USA). The settings on the growth chamber were 12-hour light/12-hour dark with light (110 μmol m^{-2} s^{-1}), CO_2 levels of ~600 ppm, 55 to 65% relative humidity, and a temperature of 21°C. Plants were 6 to 8 weeks old. Analyses were performed using a portable gas exchange system (LI-6400 and LI-6400XT, LI-COR, Lincoln, NE, USA) with the light-emitting diode light source set to 150 μmol m^{-2} s^{-1}. The relative humidity for experiments was kept between 60 to 70% with the air flow set to 400 μmol s^{-1} and the leaf temperature set to 21°C. Before the carbon dioxide experiments began, each leaf was left to acclimate to ambient CO_2 levels (~400 ppm) until the stomatal conductance was stable. For Figs. 1E and 6B, T2 plants showing clear green fluorescent protein (GFP) fluorescence in guard cells were directly analyzed by gas exchange analyses.

For fig. S4, stomatal conductances were analyzed in intact leaves of 6- to 8-week-old plants grown under 70 to 80% relative air humidity and 12-hour/12-hour light cycles using the LI-6800 Portable Photosynthesis System with an integrated multiphase flash fluorometer (6800-01A, LI-COR Inc.). Gas exchange analyses were started from 1 to 2 hours after growth chamber light onset every day. Leaves were clamped and kept at 400 ppm ambient CO_2, 21°C heat exchanger temperature, 68% relative air humidity, red light (450 μmol m^{-2} s^{-1}) combined with blue light (50 μmol m^{-2} s^{-1}), and incoming airflow rate (500 μmol s^{-1}) for 1.5 to 2 hours until stomatal conductance stabilized. For stomatal responses to [CO_2] shifts, stomatal conductance was first measured at 400 ppm ambient CO_2; then, CO_2 concentration was shifted to 900 ppm and then changed to 100 ppm as shown in the figure. Average stomatal conductances and SEs at the corresponding time points were determined from the leaves of independent plants in each genotype.

Isolation of HT1 mutants in genetic screen

The six *ht1* mutants were isolated in an O_3-sensitivity and stomatal function mutant screen (*17*) where sensitivity to O_3 was used as a proxy for more open stomata or impaired O_3-induced stomatal closure. *Arabidopsis* plants expressing *pGC1::YC3.6* (*15*) were mutagenized with 0.4% ethyl methanesulfonate as described (*17*, *36*). Two-week-old M2 plants were treated with O_3 [6 hours, 275 to 350 parts per billion (ppb)], individual rosettes displaying visible lesions were imaged 1 to 2 weeks later using a thermal camera, and water loss after 2 hours was measured from detached leaves. Phenotypes were reconfirmed in the M3 generation before selecting lines for gas exchange measurements, where the stomatal CO_2 response was analyzed. Ozone sensitivity and water loss from detached leaves were assessed in the progeny of the selected plants to isolate lines with pronounced phenotypes that may be linked to stomatal responses. In the next phase, stomatal responses to elevated CO_2 were measured with a whole-plant gas exchange system (PlantInvent Ltd.) (*37*). A total of 551 plant lines with impaired stomatal functioning were identified. Subsequently, genes known to affect stomatal functioning were sequenced in the most pronounced lines. This led to the identification of six lines carrying point mutations in *HT1*, including *ht1-G89R*, *ht1-R173Q*, and four *ht1-A109V* lines, which were backcrossed to the initial line five times before further gas exchange analyses.

Whole-plant gas exchange analyses

Experiments were performed as described before (*23*). Plants were grown in 2:1 peat:vermiculate mix with 12-hour light/12-hour dark cycles, 23°C/18°C at day/night, 70% air humidity, and light intensity (130 μmol m^{-2} s^{-1}). For whole-plant gas exchange analysis, 23- to 27-day-old plants were used. Measurements of stomatal conductance were carried out with a temperature-controlled multicuvette gas exchange device (PlantInvent Ltd.). Plants were inserted into gas exchange cuvettes and allowed to acclimate for ~1 hour at 70% humidity, 24°C, light intensity (150 μmol m^{-2} s^{-1}), and 400 ppm CO_2. After stomatal conductance stabilization, CO_2 concentration was increased to 800 ppm, then reduced to 400 ppm, and later to 100 ppm as indicated.

BiFC experiments

Nicotiana benthamiana plants were grown in standard potting soil (Sungrow Horticulture, Professional Growing Mix, MA, USA) under long-day conditions (16-hour light/8-hour dark cycle, 22°C) and 60% relative air humidity. For low CO_2 experiments, plants grown under the above conditions were exposed to low CO_2 for 2 hours before infiltration and then incubated in a growth chamber (Percival, IntellusUltra AR-41L2) after infiltration, under low CO_2 conditions (100 ppm) for 3 days under constant light conditions at 22°C and 60% relative air humidity. For high

CO_2 experiments, the low CO_2–treated plants were subjected to elevated CO_2 in a growth chamber for 4 hours to 800 ppm CO_2. During microscopy of low CO_2 samples, plants were gassed continuously with low CO_2 by passing filtered air through CO_2 absorbent to maintain the CO_2 concentration close to nominally 100 ppm.

For BiFC experiments, the coding sequences (CDSs) of HT1, MPK4, and MPK12 were cloned into vectors pDEST-VYNE(R)^{GW} (HT1) and pDEST-VYCE(R)^{GW} (MPK4 and MPK12) (*38*). All constructs were expressed under 35*S* promoter. All the constructs were sequence-verified and then transformed to *Agrobacterium tumefaciens* (GV3101 strain) for BiFC experiments. For transient expression, the *A. tumefaciens* strain harboring the BiFC constructs were used along with the p19 strain for infiltration of 5- to 6-week-old leaves of *N. benthamiana*. The combinations of constructs, nVenus-HT1 and cVenus-MPK4 or cVenus-MPK12, nVenus-HT1 and cVenus-empty vector (negative control), and nVenus-ABI1 and cVenus-GEF1 (positive control) were used for infiltrations. The innoculants were blinded by a noncoauthor/noncollaborating laboratory member and only unblinded for a third laboratory member after all analyses of blinded data were completed by the experimenter and after the blinded data had been sent to the third laboratory member. For the BiFC constructs, the strains were infiltrated at an OD_{600} of 0.5 and at an OD_{600} of 0.3 for the p19 strain for each clone in the infiltration buffer [10 mM MES (pH 5.6), 10 mM $MgCl_2$, and 200 μl acetosyringone] (*39*). Microscopy was performed 3 days after infiltration with a Nikon Eclipse E600 fluorescence microscope using a 20× objective lens with an attached INFINITYX digital charge-coupled device color microscopy camera. The Venus signals were excited by 515 nm, and emission between 528 nm was collected. For each leaf, images from nonoverlapping regions were captured. For each image, four points from nonoverlapping areas were analyzed. Images were obtained and are shown using constant imaging conditions, e.g., magnification, exposure time, gain, and offset. The fluorescence intensity of the images was measured using ImageJ software.

Coimmunoprecipitation assays using mesophyll cell protoplasts

Transient expression in *Arabidopsis* mesophyll cell protoplasts by the polyethylene glycol–mediated method was performed as described previously (*35*) using pUC18 plasmids carrying *35S:GFP:nosT*, *35S:MPK4-GFP:nosT*, and *35S:HT1-FLAG:nosT*. Protoplasts were incubated with 20 mM $NaHCO_3$ or NaCl (control) for 30 min at room temperature in 400 μl of incubation buffer [10 mM MES-KOH (pH 6.0), 0.4 M mannitol, 20 mM KCl, and 1 mM $CaCl_2$] and collected by a centrifugation for 3 min at 100*g*. After removing 200 μl of supernatant, 200 μl of 2× protein extraction buffer [100 mM MOPS-KOH (pH 7.5), 5 mM EDTA, 200 mM NaCl, 0.2% Triton X-100, 20 mM NaF, 2 mM dithiothreitol, 2 mM phenylmethylsulfonyl fluoride, and 200 μM leupeptin] supplemented with 20 mM $NaHCO_3$ or NaCl was added and incubated for 15 min on ice. After centrifugation at 14,000*g* for 10 min, resulting supernatants were transferred to new test tubes. Eight microliters of supernatants was placed on ice for input samples during immunoprecipitation. The rest of supernatants were mixed with polyclonal GFP antibodies bound to Dynabeads protein G and incubated for 60 min at 4°C with gentle mixing. After washing the beads with 1 ml of T-TBS supplemented with 20 mM $NaHCO_3$ or NaCl three times, 25 μl of SDS-PAGE loading buffer was added and incubated at 95°C for 3 min.

Structure prediction with AlphaFold2

Protein structure prediction was completed with AlphaFold2 (*26*) using the monomer or the multimer (*27*) functionality, depending on whether the prediction was for a single protein or for a protein complex, respectively. We downloaded the source code from the AlphaFold2 Github page (https://github.com/deepmind/alphafold). Each protein structure prediction was for that protein found in *Arabidopsis thaliana*. We predicted the complex of the long form of HT1 (UniProt ID: Q2MHE4) separately with MPK4 (UniProt ID: Q39024) and MPK12 (UniProt ID: Q8GYQ5). We also predicted the uncomplexed structure of HT1 and MPK12 using the same UniProt ID numbers as above. The maximum template release date that we used was from 14 May 2020. We used the full genetic database configuration and included a final relaxation step on all predicted models. For the complex predictions only, we also used five predictions per complex, each starting with a random seed. Apart from the maximum template release date, which must be set manually, all of these are the default settings from AlphaFold2. This structure prediction workflow outputs five structures ranked by their predicted template modeling (pTM) score; we selected the top-ranked structure in each case, even if this structure had a slightly lower predicted local difference distance test (pLDDT) score than a model that ranked lower in the pTM ranking. We used the pLDDT to predict which regions of the protein or protein complex are disordered and used the predicted aligned error to measure which regions of the protein were predicted with high confidence.

Gaussian accelerated molecular dynamics

GaMD (*28*–*30*) simulations were performed on the top-ranked HT1-MPK12 complex, as ranked by the pTM score (this is the default AlphaFold2 ranking system). By default, AlphaFold2 performs a restrained minimization using AMBER99FFSB (*40*) and a full minimization using OpenMM 7 (*41*). Protonation is also done with OpenMM 7 at pH 7.0. Full details can be found in the original AlphaFold2 paper (*26*). This leaves the system with an overall charge of +13. To reduce the charge to neutral and to match the 0.15 M NaCl solution used in the experimental buffer, 84 Cl^- and 71 Na^+ ions were added after consulting the screening layer tally by container average potential calculator (*42*) and confirming this methodology elsewhere (*43*). The experimental buffer contained 0.05 M tris-HCl, 0.15 M NaCl, 0.1% Triton X-100, and 1 mM DTT, which was approximated with 0.15 M NaCl. Although the experimental work was completed at pH 7.4 and AlphaFold2 protonates structures at pH 7.0, we estimate that this discrepancy would not affect our results. We added the 0.15 M NaCl through Amber's tleap module (*44*) using the CUDA version 10.1 implementation (*45*–*47*) of Amber 20 (*44*). The OPC water model (*48*) was used in conjunction with the Amber19ffsb force field (*49*). This system contained 140,866 atoms. This solvated system was then minimized again for 10,000 cycles: 1000 cycles of steepest descent, followed by 9000 cycles of conjugate gradient (*45*). The heavy atoms were restrained with a force constant of 1.0 kcal/(mol × Å^2). The system was then slowly heated from 10.0 to 300.0 K over the course of 4 ns before plateauing at 300.0 K for the following 6 ns in the NVT ensemble with a Langevin thermostat (*50*, *51*)

containing a friction coefficient (collision frequency) of γ = 5.0 ps^{-1}. During the heating, the heavy atoms were restrained with a force constant of 1.0 kcal/(mol × $Å^2$). Next, equilibration was performed in the NPT ensemble for 10 ns, with a time step of 2 fs. The SHAKE algorithm was used to constrain bonds involving hydrogen (*52*). The equilibration temperature was 300.0 K with a Langevin thermostat containing a friction coefficient (collision frequency) of γ = 1.0 ps^{-1}. The heavy atoms were restrained with a force constant of 0.1 kcal/(mol × $Å^2$). Periodic boundary conditions were set in place with a van der Waals interaction cutoff of 8 Å, and the long range interactions were treated with the particle mesh Ewald algorithm (*53*). The pressure was treated with a Berendsen barostat (*54*) and was set to 1 bar. The relaxation time constant was τ = 1.0 ps^{-1}. This structure was then cloned into five replicates. Each replicate separately underwent a GaMD equilibration starting with its own random seed. During the GaMD equilibration, the bonds involving hydrogen were again treated with SHAKE. The equilibration was performed in the NVT ensemble, at a temperature of 300.0 K and with a Langevin thermostat. A GaMD dual boost on both the dihedral and the total potential energy was applied to the system. The upper limits of the SD of the first and second potential boosts were 6.0 kcal/mol each, which is the default. The threshold energy was set to be $E = V_{max}$, which is the default. GaMD equilibration necessitates a number of conventional MD steps to measure the potential energies; we used 0.4 ns of molecular dynamics (MD) prep (where potential energy statistics are not collected), followed by 2 ns of conventional MD (where potential energy statistics are collected). Next, for the GaMD prep, the equilibration added the boost potential for the next 1.6 ns but did not update the potential energy statistics. Last, we updated the potential energy statistics and ran 50 ns of biasing MD steps with these updated statistics. We set the total amount of equilibration time to be 52 ns; this can be done as the MD prep and the GaMD prep can be incorporated into their following, longer equilibration times, and the acceleration parameters/potential energy statistics are adaptively updated. With the GaMD equilibration done, we ran 500 ns of GaMD on each of the five replicates.

Clustering

The complex trajectories were concatenated, aligned to the residue heavy atom backbones (the two carbons and the nitrogen), and clustered into three states using Gromacs (*55*), specifically with the Gromos method (*56*) with a cutoff of 6.5 Å, resulting in three clusters.

Mutation prediction

The top clustered state from the GaMD simulations, as described above, was used for predicting mutations that would interfere with the protein-protein interface. First, this structure was put through a residue scan on the interface residues in Molecular Operating Environment (MOE) 2020 (*57*) using the Amber10:EHT force field (*58*, *59*) to determine which mutations would most interfere with the protein-protein interface. The interface residues were determined with MOE. We selected the top 10 mutations and put these through three further scans, each using a different methodology, to try to find a consensus for which initial mutation should be tested experimentally. The first program was GeoPPI (*60*), the second was Mutabind2 (*61*), and the third was the LowModeMD functionality of MOE (*62*), each using the default parameters. From these scans, we selected the MPK12-Y277G mutation for initial experimental testing. Simultaneously, we also used the G89R, R102K, A109V, and R173Q (all on HT1) mutations through all four scans (GeoPPI, Mutabind2, MOE residue scan, and MOE LowModeMD scan) as reference.

Supplementary Materials

This PDF file includes:
Figs. S1 to S9
Tables S1 to S3

View/request a protocol for this paper from *Bio-protocol*.

REFERENCES AND NOTES

1. E. A. Ainsworth, S. P. Long, What have we learned from 15 years of free-air CO_2 enrichment (FACE)? A meta-analytic review of the responses of photosynthesis, canopy properties and plant production to rising CO_2. *New Phytol.* **165**, 351–372 (2005).
2. A. M. Hetherington, F. I. Woodward, The role of stomata in sensing and driving environmental change. *Nature* **424**, 901–908 (2003).
3. J. Negi, M. Hashimoto-Sugimoto, K. Kusumi, K. Iba, New approaches to the biology of stomatal guard cells. *Plant Cell Physiol.* **55**, 241–250 (2014).
4. Z. Yang, J. Liu, S. V. Tischer, A. Christmann, W. Windisch, H. Schnyder, E. Grill, Leveraging abscisic acid receptors for efficient water use in *Arabidopsis*. *Proc. Natl. Acad. Sci. U.S.A.* **113**, 6791–6796 (2016).
5. J. Zhang, P. De-Oliveira-Ceciliato, Y. Takahashi, S. Schulze, G. Dubeaux, F. Hauser, T. Azoulay-Shemer, K. Tõldsepp, H. Kollist, W. J. Rappel, J. I. Schroeder, Insights into the molecular mechanisms of CO_2-mediated regulation of stomatal movements. *Curr. Biol.* **28**, R1356–R1363 (2018).
6. A. Wu, G. L. Hammer, A. Doherty, S. von Caemmerer, G. D. Farquhar, Quantifying impacts of enhancing photosynthesis on crop yield. *Nat Plants* **5**, 380–388 (2019).
7. H. Hu, A. Boisson-Dernier, M. Israelsson-Nordström, M. Böhmer, S. Xue, A. Ries, J. Godoski, J. M. Kuhn, J. I. Schroeder, Carbonic anhydrases are upstream regulators of CO_2-controlled stomatal movements in guard cells. *Nat. Cell Biol.* **12**, 87–93 (2010).
8. A. R. Kolbe, T. P. Brutnell, A. B. Cousins, A. J. Studer, Carbonic anhydrase mutants in *Zea mays* have altered stomatal responses to environmental signals. *Plant Physiol.* **177**, 980–989 (2018).
9. S. Xue, H. Hu, A. Ries, E. Merilo, H. Kollist, J. I. Schroeder, Central functions of bicarbonate in S-type anion channel activation and OST1 protein kinase in CO_2 signal transduction in guard cell. *EMBO J.* **30**, 1645–1658 (2011).
10. J. Zhang, N. Wang, Y. Miao, F. Hauser, J. A. McCammon, W. J. Rappel, J. I. Schroeder, Identification of SLAC1 anion channel residues required for CO_2/bicarbonate sensing and regulation of stomatal movements. *Proc. Natl. Acad. Sci. U.S.A.* **115**, 11129–11137 (2018).
11. T. Kinoshita, S. Toh, K. U. Torii, Chemical control of stomatal function and development. *Curr. Opin. Plant Biol.* **60**, 102010 (2021).
12. M. Hashimoto, J. Negi, J. Young, M. Israelsson, J. I. Schroeder, K. Iba, *Arabidopsis* HT1 kinase controls stomatal movements in response to CO_2. *Nat. Cell Biol.* **8**, 391–397 (2006).
13. A. Hiyama, A. Takemiya, S. Munemasa, E. Okuma, N. Sugiyama, Y. Tada, Y. Murata, K. I. Shimazaki, Blue light and CO_2 signals converge to regulate light-induced stomatal opening. *Nat. Commun.* **8**, 1284 (2017).
14. K. Tõldsepp, J. Zhang, Y. Takahashi, Y. Sindarovska, H. Hõrak, P. H. O. Ceciliato, K. Koolmeister, Y. S. Wang, L. Vaahtera, L. Jakobson, C. Y. Yeh, J. Park, M. Brosche, H. Kollist, J. I. Schroeder, Mitogen-activated protein kinases MPK_4 and MPK12 are key components mediating CO_2-induced stomatal movements. *Plant J.* **96**, 1018–1035 (2018).
15. Y. Yang, A. Costa, N. Leonhardt, R. S. Siegel, J. I. Schroeder, Isolation of a strong *Arabidopsis* guard cell promoter and its potential as a research tool. *Plant Methods* **4**, 6 (2008).
16. D. Hua, C. Wang, J. He, H. Liao, Y. Duan, Z. Zhu, Y. Guo, Z. Chen, Z. Gong, A plasma membrane receptor kinase, GHR1, mediates abscisic acid- and hydrogen peroxide-regulated stomatal movement in *Arabidopsis*. *Plant Cell* **24**, 2546–2561 (2012).
17. M. Sierla, H. Horak, K. Overmyer, C. Waszczak, D. Yarmolinsky, T. Maierhofer, J. P. Vainonen, J. Salojarvi, K. Denessiouk, K. Laanemets, K. Toldsepp, T. Vahisalu, A. Gauthier, T. Puukko, L. Paulin, P. Auvinen, D. Geiger, R. Hedrich, H. Kollist, J. Kangasjarvi, The receptor-like pseudokinase GHR1 is required for stomatal closure. *Plant Cell* **30**, 2813–2837 (2018).
18. M. J. Cann, A. Hammer, J. Zhou, T. Kanacher, A defined subset of adenylyl cyclases is regulated by bicarbonate ion. *J. Biol. Chem.* **278**, 35033–35038 (2003).

19. Y. Chen, M. J. Cann, T. N. Litvin, V. Iourgenko, M. L. Sinclair, L. R. Levin, J. Buck, Soluble adenylyl cyclase as an evolutionarily conserved bicarbonate sensor. *Science* **289**, 625–628 (2000).
20. P. D. Townsend, P. M. Holliday, S. Fenyk, K. C. Hess, M. A. Gray, D. R. Hodgson, M. J. Cann, Stimulation of mammalian G-protein-responsive adenylyl cyclases by carbon dioxide. *J. Biol. Chem.* **284**, 784–791 (2009).
21. L. Jakobson, L. Vaahtera, K. Toldsepp, M. Nuhkat, C. Wang, Y. S. Wang, H. Horak, E. Valk, P. Pechter, Y. Sindarovska, J. Tang, C. Xiao, Y. Xu, U. Gerst Talas, A. T. Garcia-Sosa, S. Kangasjarvi, U. Maran, M. Remm, M. R. Roelfsema, H. Hu, J. Kangasjarvi, M. Loog, J. I. Schroeder, H. Kollist, M. Brosche, Natural variation in *Arabidopsis* Cvi-0 accession reveals an important role of MPK12 in guard cell CO_2 signaling. *PLoS Biol.* **14**, e2000322 (2016).
22. T. Vahisalu, H. Kollist, Y. F. Wang, N. Nishimura, W. Y. Chan, G. Valerio, A. Lamminmaki, M. Brosche, H. Moldau, R. Desikan, J. I. Schroeder, J. Kangasjarvi, SLAC1 is required for plant guard cell S-type anion channel function in stomatal signalling. *Nature* **452**, 487–491 (2008).
23. H. Hõrak, M. Sierla, K. Tõldsepp, C. Wang, Y. S. Wang, M. Nuhkat, E. Valk, P. Pechter, E. Merilo, J. Salojärvi, K. Overmyer, M. Loog, M. Brosché, J. I. Schroeder, J. Kangasjärvi, H. Kollist, A dominant mutation in the HT1 kinase uncovers roles of MAP kinases and GHR1 in CO_2-induced stomatal closure. *Plant Cell* **28**, 2493–2509 (2016).
24. M. Hashimoto-Sugimoto, J. Negi, K. Monda, T. Higaki, Y. Isogai, T. Nakano, S. Hasezawa, K. Iba, Dominant and recessive mutations in the Raf-like kinase HT1 gene completely disrupt stomatal responses to CO_2 in *Arabidopsis*. *J. Exp. Bot.* **67**, 3251–3261 (2016).
25. Z. Li, Y. Takahashi, A. Scavo, B. Brandt, D. Nguyen, P. Rieu, J. I. Schroeder, Abscisic acid-induced degradation of *Arabidopsis* guanine nucleotide exchange factor requires calcium-dependent protein kinases. *Proc. Natl. Acad. Sci. U.S.A.* **115**, E4522–E4531 (2018).
26. J. Jumper, R. Evans, A. Pritzel, T. Green, M. Figurnov, O. Ronneberger, K. Tunyasuvunakool, R. Bates, A. Žídek, A. Potapenko, A. Bridgland, C. Meyer, S. A. A. Kohl, A. J. Ballard, A. Cowie, B. Romera-Paredes, S. Nikolov, R. Jain, J. Adler, T. Back, S. Petersen, D. Reiman, E. Clancy, M. Zielinski, M. Steinegger, M. Pacholska, T. Berghammer, S. Bodenstein, D. Silver, O. Vinyals, A. W. Senior, K. Kavukcuoglu, P. Kohli, D. Hassabis, Highly accurate protein structure prediction with AlphaFold. *Nature* **596**, 583–589 (2021).
27. R. Evans, M. O'Neill, A. Pritzel, N. Antropova, A. Senior, T. Green, A. Žídek, R. Bates, S. Blackwell, J. Yim, O. Ronneberger, S. Bodenstein, M. Zielinski, A. Bridgland, A. Potapenko, A. Cowie, K. Tunyasuvunakool, R. Jain, E. Clancy, *P. Kohli*, J. Jumper, D. Hassabis, Protein complex prediction with AlphaFold-Multimer. bioRxiv 2021.10.04.463034 [Preprint]. 10 March 2022.https://doi.org/10.1101/2021.10.04.463034.
28. Y. Miao, V. A. Feher, J. A. McCammon, Gaussian accelerated molecular dynamics: Unconstrained enhanced sampling and free energy calculation. *J. Chem. Theory Comput.* **11**, 3584–3595 (2015).
29. A. Bhattarai, Gaussian accelerated molecular dynamics for elucidation of drug pathways. *Expert Opin. Drug Discov.* **13**, 1055–1065 (2018).
30. Y. Miao, Acceleration of biomolecular kinetics in Gaussian accelerated molecular dynamics. *J. Chem. Phys.* **149**, 072308 (2018).
31. D. L. Des Marais, L. C. Auchincloss, E. Sukamtoh, J. K. McKay, T. Logan, J. H. Richards, T. E. Juenger, Variation in *MPK12* affects water use efficiency in *Arabidopsis* and reveals a pleiotropic link between guard cell size and ABA response. *Proc. Natl. Acad. Sci. U.S.A.* **111**, 2836–2841 (2014).
32. B. E. Campitelli, D. L. Des Marais, T. E. Juenger, Ecological interactions and the fitness effect of water-use efficiency: Competition and drought alter the impact of natural MPK12 alleles in *Arabidopsis*. *Ecol. Lett.* **19**, 424–434 (2016).
33. G. J. Dow, D. C. Bergmann, J. A. Berry, An integrated model of stomatal development and leaf physiology. *New Phytol.* **201**, 1218–1226 (2014).
34. N. M. L. Simon, C. A. Graham, N. E. Comben, A. M. Hetherington, A. N. Dodd, The circadian clock influences the long-term water use efficiency of *Arabidopsis*. *Plant Physiol.* **183**, 317–330 (2020).
35. Y. Takahashi, J. Zhang, P. K. Hsu, P. H. O. Ceciliato, L. Zhang, G. Dubeaux, S. Munemasa, C. Ge, Y. Zhao, F. Hauser, J. I. Schroeder, MAP3Kinase-dependent SnRK2-kinase activation is required for abscisic acid signal transduction and rapid osmotic stress response. *Nat. Commun.* **11**, 12 (2020).
36. Y. Kim, K. S. Schumaker, J. K. Zhu, EMS mutagenesis of *Arabidopsis*. *Methods Mol. Biol.* **323**, 101–103 (2006).
37. T. Kollist, H. Moldau, B. Rasulov, V. Oja, H. Rämma, K. Hüve, P. Jaspers, J. Kangasjärvi, H. Kollist, A novel device detects a rapid ozone-induced transient stomatal closure in intact *Arabidopsis* and its absence in abi2 mutant. *Physiol. Plant.* **129**, 796–803 (2007).
38. C. Gehl, R. Waadt, J. Kudla, R. R. Mendel, R. Hansch, New GATEWAY vectors for high throughput analyses of protein-protein interactions by bimolecular fluorescence complementation. *Mol. Plant* **2**, 1051–1058 (2009).
39. R. Waadt, L. K. Schmidt, M. Lohse, K. Hashimoto, R. Bock, J. Kudla, Multicolor bimolecular fluorescence complementation reveals simultaneous formation of alternative CBL/CIPK complexes in planta. *Plant J.* **56**, 505–516 (2008).
40. J. Wang, P. Cieplak, P. A. Kollman, How well does a restrained electrostatic potential (RESP) model perform in calculating conformational energies of organic and biological molecules? *J. Comput. Chem.* **21**, 1049–1074 (2000).
41. P. Eastman, J. Swails, J. D. Chodera, R. T. McGibbon, Y. Zhao, K. A. Beauchamp, L.-P. Wang, A. C. Simmonett, M. P. Harrigan, C. D. Stern, R. P. Wiewiora, B. R. Brooks, V. S. Pande, OpenMM 7: Rapid development of high performance algorithms for molecular dynamics. *PLoS Comput. Biol.* **13**, e1005659 (2017).
42. J. D. Schmit, N. L. Kariyawasam, V. Needham, P. E. Smith, SLTCAP: A simple method for calculating the number of ions needed for MD simulation. *J. Chem. Theory Comput.* **14**, 1823–1827 (2018).
43. M. R. Machado, S. Pantano, Split the charge difference in two! A rule of thumb for adding proper amounts of ions in MD simulations. *J. Chem. Theory Comput.* **16**, 1367–1372 (2020).
44. D. A. Case, H. M. Aktulga, K. Belfon, I. Y. Ben-Shalom, S. R. Brozell, D. S. Cerutti, I. Thomas E. Cheatham, G. A. Cisneros, V. W. D. Cruzeiro, T. A. Darden, R. E. Duke, G. Giambasu, M. K. Gilson, H. Gohlke, A. W. Götz, R. Harris, S. Izadi, S. A. Izmailov, C. Jin, K. Kasavajhala, M. C. Kaymak, E. King, A. Kovalenko, T. Kurtzman, T. S. Lee, S. LeGrand, P. Li, C. Lin, J. Liu, T. Luchko, R. Luo, M. Machado, V. Man, M. Manathunga, K. M. Merz, Y. Miao, O. Mikhailovskii, G. Monard, H. Nguyen, K. A. O'Hearn, A. Onufriev, F. Pan, S. Pantano, R. Qi, A. Rahnamoun, D. R. Roe, A. Roitberg, *C. Sagui*, S. Schott-Verdugo, J. Shen, C. L. Simmerling, N. R. Skrynnikov, J. Smith, J. Swails, R. C. Walker, J. Wang, H. Wei, R. M. Wolf, X. Wu, Y. Xue, D. M. York, S. Zhao, P. A. Kollman, AMBER 2020 (University of California, San Francisco, 2020).
45. R. Salomon-Ferrer, A. W. Götz, D. Poole, S. LeGrand, R. C. Walker, Routine microsecond molecular dynamics simulations with AMBER on GPUs. 2. Explicit solvent particle mesh Ewald. *J. Chem. Theory Comput.* **9**, 3878–3888 (2013).
46. A. W. Götz, M. J. Williamson, D. Xu, D. Poole, S. L. Grand, R. C. Walker, Routine microsecond molecular dynamics simulations with AMBER on GPUs. 1. Generalized born. *J. Chem. Theory Comput.* **8**, 1542–1555 (2012).
47. S. L. Grand, A. W. Götz, R. C. Walker, SPFP: Speed without compromise—A mixed precision model for GPU accelerated molecular dynamics simulations. *Comput. Phys. Commun.* **184**, 374–380 (2013).
48. S. Izadi, R. Anandakrishnan, A. V. Onufriev, Building water models: A different approach. *J. Phys. Chem. Lett.* **5**, 3863–3871 (2014).
49. C. Tian, K. Kasavajhala, K. A. A. Belfon, L. Raguette, H. Huang, A. N. Migues, J. Bickel, Y. Wang, J. Pincay, Q. Wu, C. Simmerling, ff19SB: Amino-acid-specific protein backbone parameters trained against quantum mechanics energy surfaces in solution. *J. Chem. Theory Comput.* **16**, 528–552 (2020).
50. R. J. Loncharich, B. R. Brooks, R. W. Pastor, Langevin dynamics of peptides: The frictional dependence of isomerization rates of *N*-acetylalanyl-*N*′-methylamide. *Biopolymers* **32**, 523–535 (1992).
51. R. W. Pastor, B. R. Brooks, A. Szabo, An analysis of the accuracy of Langevin and molecular dynamics algorithms. *Mol. Phys.* **65**, 1409–1419 (1988).
52. J.-P. Ryckaert, G. Ciccotti, H. J. C. Berendsen, Numerical integration of the cartesian equations of motion of a system with constraints: Molecular dynamics of *n*-alkanes. *J. Comput. Phys.* **23**, 327–341 (1977).
53. T. Darden, D. M. York, L. G. Pedersen, Particle mesh Ewald: AnN·log(N) method for Ewald sums in large systems. *J. Chem. Phys.* **98**, 10089–10092 (1993).
54. H. J. C. Berendsen, J. P. M. Postma, W. F. V. Gunsteren, A. D. Nola, J. R. Haak, Molecular dynamics with coupling to an external bath. *J. Chem. Phys.* **81**, 3684–3690 (1984).
55. M. J. Abraham, T. Murtola, R. Schulz, S. Páll, J. C. Smith, B. Hess, E. Lindahl, GROMACS: High performance molecular simulations through multi-level parallelism from laptops to supercomputers. *SoftwareX* **1-2**, 19–25 (2015).
56. X. Daura, K. Gademann, B. Jaun, D. Seebach, W. F. V. Gunsteren, A. E. Mark, Peptide folding: When simulation meets experiment. *Angew. Chem. Int. Ed.* **38**, 236–240 (1999).
57. Molecular Operating Environment (MOE), Release 2020 (1010 Sherbooke St. West, Suite #910, Montreal, QC, Canada, H3A 2R7, 2020).
58. D. A. Case, T. Darden, T. E. C. III, C. Simmerling, J. Wang, R. E. Duke, R. Luo, M. Crowley, R. Walker, W. Zhang, K. M. Merz, B. Wang, S. Hayik, A. Roitberg, G. Seabra, I. Kolossváry, K. F. Wong, F. Paesani, J. Vanicek, X. Wu, S. R. Brozell, T. Steinbrecher, H. Gohlke, L. Yang, C. Tan, J. Mongan, V. Hornak, *G. Cui*, D. H. Mathews, M. G. Seetin, *C. Sagui*, V. Babin, P. A. Kollman, AMBER 10 (University of California, San Francisco, 2008).
59. P. R. Gerber, K. Müller, MAB, a generally applicable molecular force field for structure modelling in medicinal chemistry. *J. Comput. Aided Mol. Des.* **9**, 251–268 (1995).
60. X. Liu, Y. Luo, P. Li, S. Song, J. Peng, Deep geometric representations for modeling effects of mutations on protein-protein binding affinity. *PLoS Comput. Biol.* **17**, e1009284 (2021).

61. N. Zhang, Y. Chen, H. Lu, F. Zhao, R. V. Alvarez, A. Goncearenco, A. R. Panchenko, MinghuiLi, MutaBind2: Predicting the impacts of single and multiple mutations on protein-protein interactions. *iScience* **23**, 100939 (2020).
62. P. Labute, LowModeMD—Implicit low-mode velocity filtering applied to conformational search of macrocycles and protein loops. *J. Chem. Inf. Model.* **50**, 792–800 (2010).

Acknowledgments: We thank K. Shimazaki (Kyushu University) for providing *cbc1 cbc2* double-mutant seeds. **Funding:** This research was funded by the National Science Foundation grant MCB-1900567 to J.I.S. and in part supported by the National Institutes of Health grant R01 GM60396 to J.I.S. Y.T. was supported by JST, PRESTO grant number JPMJPR21D8, and by a SUNBOR grant. Genetic isolation and initial stomatal conductance measurements of two of the dominant *ht1* alleles (Fig. 3, A and B, and fig. S3) were funded by Estonian Research Council grant PRG433 (to H.K.), the Centre of Excellence CEMCE (to H.K.), the Plant Biology Infrastructure project TAIM (to. H.K.), the Ella and Georg Ehrnrooth Foundation (to M.S.), an Academy of Finland grant (266793) (to T.V.), and the Academy of Finland Centre of Excellence program (2014–2019, grant numbers 271832 and 307335) (to J.K.) with screening contributions from Cezary Waszczak. C.S. was supported by a National Science Foundation Graduate Research Fellowship (DGE-1650112). J.A.M. was supported by the National Institutes of Health grant (GM31749). **Author contributions:** Y.T. and J.I.S. conceived of the project. Y.T. and J.I.S. designed research. Y.T., K.C.B., P.-K.H., K.P., C.S., C.-Y.Y., Y.-S.W., D.Y., M.S., T.V., L.Z., and T.T. performed experiments. Y.T., K.C.B., C.S., C.-Y.Y., Y.-S.W., D.Y., M.S., T.V., J.A.M., J. K. H.K., L.Z., T.T., and J.I.S. analyzed data. Y.T., K.C.B., C.S. and J.I.S. wrote the manuscript with input from the authors. **Competing interests:** The University of California, San Diego has submitted a patent on behalf of Y.T. and J.I.S. on aspects of these findings (current patent status: pending, date: 1 September 2022, serial number: 63/403,274). The authors declare that they have no other competing interests. **Data and materials availability:** All data needed to evaluate the conclusions in the paper are present in the paper and/or the Supplementary Materials. *Arabidopsis* mutants used in this study are available from the corresponding authors upon request.

Submitted 21 April 2022
Accepted 2 November 2022
Published 7 December 2022
10.1126/sciadv.abq6161
