# Supplementary material for: Stomatal CO2/bicarbonate sensor consists of two interacting protein kinases, Raf-like HT1 and non-kinase-activity requiring MPK12/MPK4
Source: Sci Adv. 2022 Dec 7;8(49):eabq6161. doi: 10.1126/sciadv.abq6161 (PMC9728965; doi:10.1126/sciadv.abq6161)
Supplement: Supplementary file 2 — Figs. S1 to S9 Tables S1 to S3 [file sciadv.abq6161_sm.pdf]

Science Advances
AAAS

Supplementary Materials for

Stomatal CO_2/bicarbonate sensor consists of two interacting protein kinases, Raf-like HT1 and non-kinase activity requiring MPK12/MPK4

Yohei Takahashi *et al.*

Corresponding author: Yohei Takahashi, ytakahashi@ucsd.edu; Julian I. Schroeder, jischroeder@ucsd.edu

Sci. Adv. **8**, eabq6161 (2022)
DOI: 10.1126/sciadv.abq6161

This PDF file includes:

Figs. S1 to S9
Tables S1 to S3

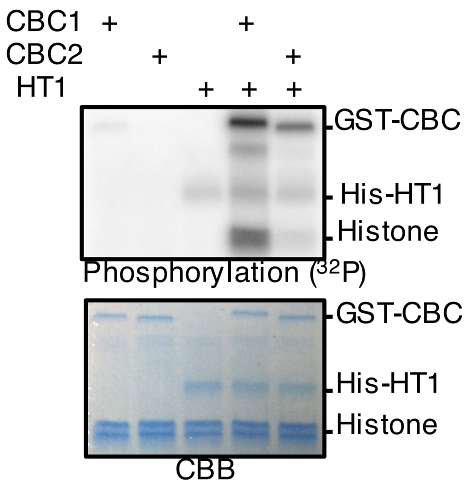

Fig. S1. The Raf-like kinase CBC proteins are phosphorylated by HT1 protein kinase.
In vitro phosphorylation analyses were performed using recombinant CBC1 and CBC2 proteins in the presence or absence of HT1. Histone was used as an artificial phosphorylation substrate of CBC1 (see main manuscript).

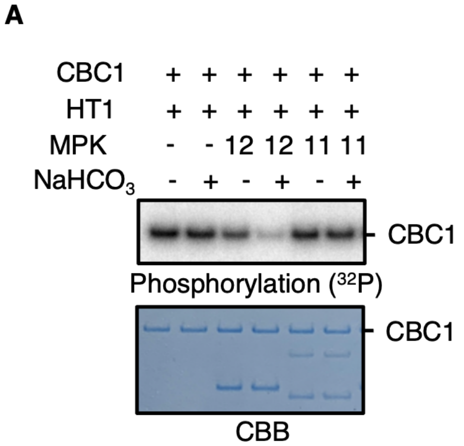

Fig. S2. MPK11 and MPK3 do not mediate $NaHCO_3$-induced inhibition of CBC1 phosphorylation by HT1 *in vitro*.

(**A**) His-HT1 and GST-CBC1 proteins were incubated with His-MPK12 or His-MPK11 protein with or without $NaHCO_3$ for 30 min, and *in vitro* phosphorylation assays were performed.

(**B**) His-HT1 and GST-CBC1 proteins were incubated with His-MPK3, His-MPK4 or His-MPK12 protein with or without $NaHCO_3$ for 30 min, and *in vitro* phosphorylation assays were performed.

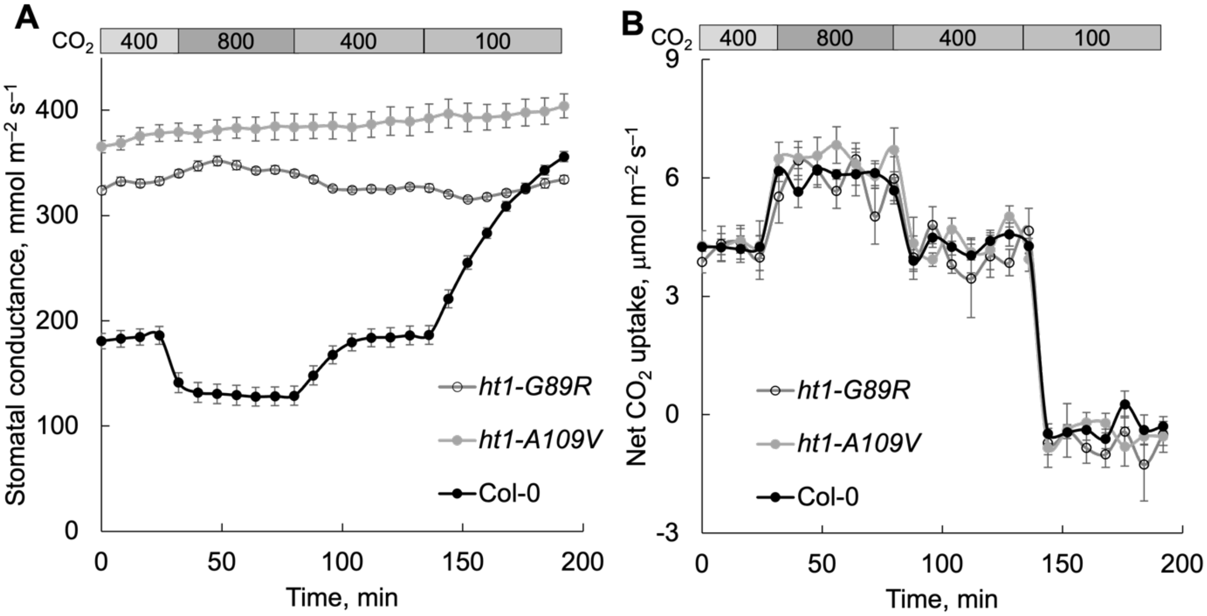

Fig. S3. Whole plant stomatal conductance measurements in the *ht1-G89R* mutant and the *ht1-A109V* mutant.

(**A**) Whole plant gas exchange analyses of Col-0, *ht1-G89R* and *ht1-A109V*. Ambient CO_2 concentrations are indicated by the top bars. n = 6 for *ht1-G89R*, n = 4 for *ht1-A109V*, and n = 4 for Col-0. Error bars show ± SEM.

(**B**) Net CO_2 uptake of plants analyzed in (A).

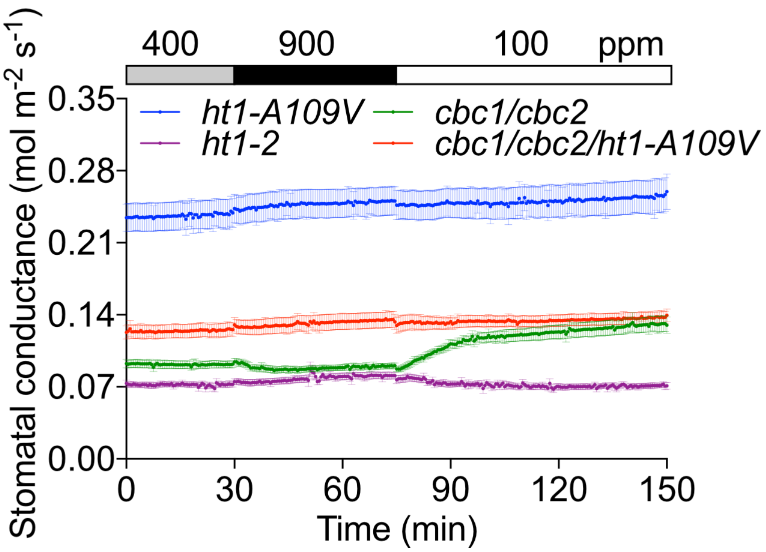

Fig. S4. ***CBC1* and *CBC2* are epistatic to *HT1*.**

Time-resolved stomatal conductance responses to ambient CO_2 concentration changes in *ht1-A109V*, *ht1-2*, *cbc1/cbc2* double, and *cbc1/cbc2/ht1-A109V* triple mutants measured with a LI-6800 gas exchange analyzer (Li-Cor Inc.). Ambient CO_2 concentrations are shown on the top of traces. n = 7 intact leaves attached to independent intact plants for each genotype. Data represent mean ± SEM.

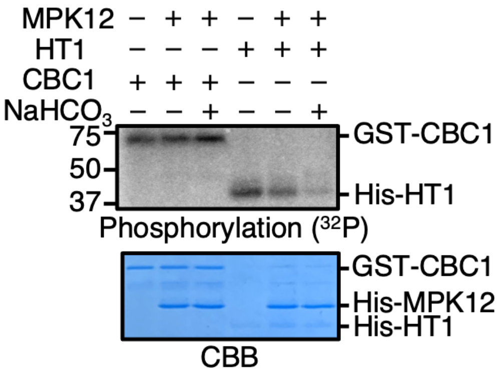

Fig. S5. Replicate example of $NaHCO_3$-dependent HT1 inhibition in the presence of MPK12. Replicate of *in vitro* phosphorylation assays using HT1 and CBC1 or MPK12 proteins with or without 20 mM $NaHCO_3$ or 20 mM NaCl ("-" controls). See Figure 4B for an independent experiment with both MPK4 and MPK12. Note that radiography gels in Figure S5 were exposed for a long period of 15 hours, in order to resolve the basal activity of the CBC1 protein kinase here.

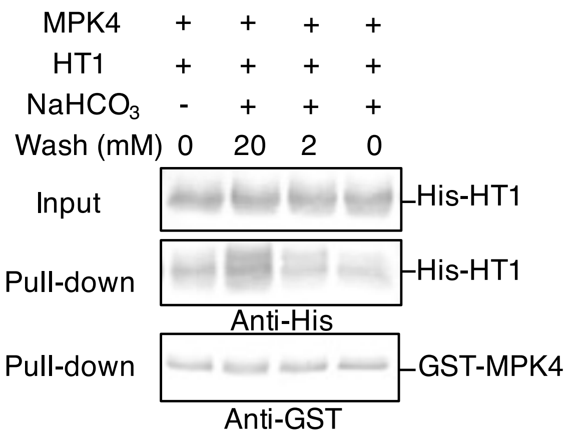

Fig. S6. Repeat experiment example of reversible interaction between MPK4 and HT1 *in vitro*.

In vitro pull-down assays using recombinant GST-MPK4 and His-HT1 proteins were performed with or without 20 mM $NaHCO_3$. Precipitated proteins were washed with buffers supplemented with $NaHCO_3$ at the indicated concentrations (0, 2 or 20 mM). See Figure 4E for an independent experiment. To control for effects of Na^+, 20 mM NaCl (0 mM $NaHCO_3$) for lanes 1 and 4 were included in the buffers; and 18 mM NaCl (2 mM $NaHCO_3$) for lane 3 were included in the buffer.

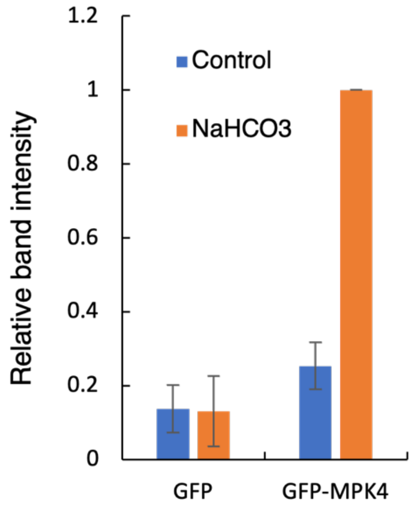

Fig. S7 Quantification of $NaHCO_3$-dependent interaction between MPK4 and HT1 in *Arabidopsis* mesophyll cell protoplasts.

Normalized co-immunoprecipitated HT1-FLAG protein band intensities as shown in Figure 4I were measured using ImageJ (version: 2.00-rc-43/1.50e), n = 3 experiments, error bars show ±s.e.m.

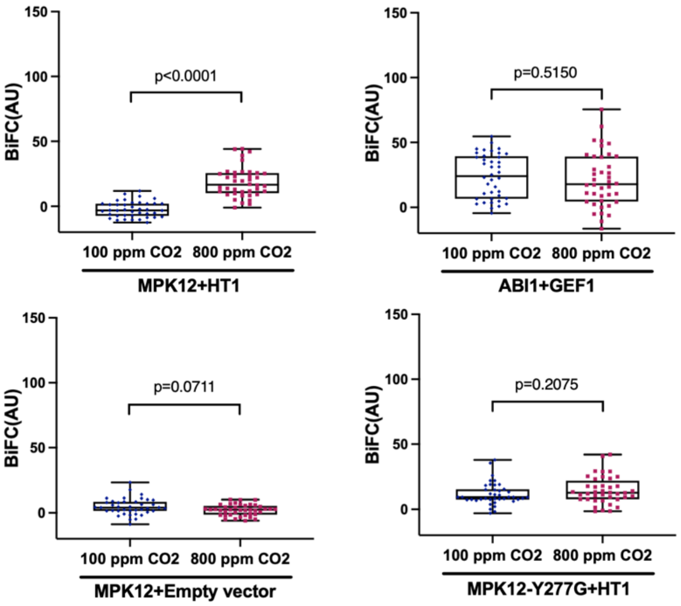

Fig. S8 The MPK12-Y277G mutation reduces CO_2-induced interaction between MPK12 and HT1 in *in planta* BiFC assays.

Beeswarm Box plots of inoculant-blinded BiFC image intensities indicates enhanced interactions of MPK12 and HT1 when plants were exposed to 800 ppm CO_2, compared to 100 ppm CO_2. In contrast, positive control ABI1 – GEF1 (25) interaction assays and MPK12 – empty vector negative controls, showed no clear difference among CO_2 concentrations. Inoculants were blinded to the experimenter and unblinded by a different person after the experimentor had analyzed all images. Data were normalized to the background autofluorescence by subtracting average control fluorescence from measured fluorescence intensities. Statistical analysis of the BiFC data was performed using paired t tests (two tailed). Ten images were analyzed for each condition and four non-overlapping areas were analyzed for each image. Note that negative fluorescence intensities reflect images in which the average background fluorescence slightly exceeded measured fluorescence intensities. All graphs and statistical analyses were performed using GraphPad Prism Software (version 9.0.0).

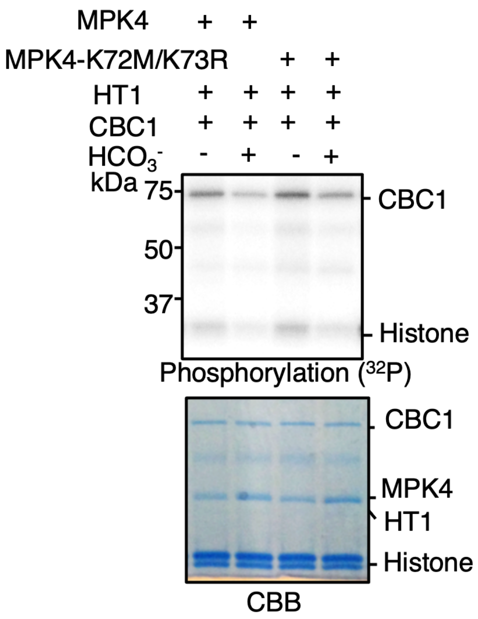

Fig. S9 Kinase-inactive MPK4 mediates $NaHCO_3$-induced inhibition of CBC1 phosphorylation.

In vitro phosphorylation assays were performed after CBC1, HT1 and MPK4 or the kinase inactive isoform (MPK12-K72M/K73R) proteins were incubated with or without $NaHCO_3$ or NaCl ("-") controls. Histone is an artificial substrate of CBC1 protein kinase (see also main text).

	Mutabind2 ΔΔG (kcal/mol)				GeoPPI ΔΔG (kcal/mol)				MOE ΔΔG (kcal/mol)			
	AF	1	2	3	AF	1	2	3	AF	1	2	3
G89R	2.02	2.29	1.32	1.14	-0.45	-0.21	-0.36	-0.16	2.42	-0.04	1.98	-0.09
R102K	1.15	0.75	1.36	0.86	1.07	0.68	0.4	-0.22	1.39	2.22	0.48	1.57
A109V	0.51	0.62	0.81	0.91	-0.55	-0.94	-0.65	-0.74	0.17	0.24	-0.09	0.03
R173Q	1.02	0.55	0.71	0.97	1.22	0.96	0.57	0.82	1.54	0.9	1.34	0.77

Table S1. Prediction of binding affinity between MPK12 and dominant mutant HT1 isoforms.

Predicted free energy change of four selected mutations in HT1 (G89R, R102K, A109V, R173Q) using three different programs (Mutabind2, GeoPPI, MOE). Positive values indicate an increased free energy (and thus a predicted destabilization of the complex). These predictions were made using the Alphafold2 structure (AF in the table) and the first, second and third cluster from the Gaussian-accelerated molecular dynamics (GaMD) simulations (noted as 1, 2, and 3, respectively) (see Methods for details).

mutation	Mutabind2	GeoPPI	MOE	lowmode	protein
	Δstability (kcal/mol)				
Y277G	2.13	1.72	3.79	4	MPK12
L83G	0.96	1.18	3.69	3.86	HT1
L213G	0.81	0.47	3.85	3.54	MPK12
L86G	0.79	1.47	4	3.46	HT1
W206G	2.31	1.9	3.68	3.45	MPK12
L281G	1.52	0.73	3.7	3.41	MPK12
Y277P	2.36	1.49	3.87	3.28	MPK12
L212G	1.55	0.96	4.47	3.24	MPK12
F6S	0.74	0.9	3.66	2.71	HT1
L212D	1.07	0.79	3.88	2.51	MPK12
R173Q	0.55	0.96	0.9	2.12	HT1
R102K	0.75	0.68	2.22	1.47	HT1
A109V	0.62	-0.94	0.24	0.15	HT1
G89R	2.29	-0.21	-0.04	-1.39	HT1

Table S2. Computational prediction of mutations that impair bicarbonate-induced MPK12–HT1 interaction. Effect on free energy of MPK12 – HT1 interaction derived from several predicted mutations of either MPK12 or HT1 using four different methods: Mutabind2, GeoPPI, MOE residue scan, and lowmode MOE. Increasing positive values indicate increasingly destabilized MPK12 – HT1 interaction. (see Methods for details).

MPK4.user.Fw	ggcttaauATGTCGGCGGAGAGTTG
MPK4.user.Rv	ggtttaauCACTGAGTCTTGAGGATTG
MPK12.user.F54	ggcttaauATGTCTGGAGAATCAAGCTC
MPK12.user.R54	ggtttaauGTGGTCAGGATTGAATTTGAC
MPK12_InF_pET_F	catggctgatatcggatccATGTCTGGAGAATCAAGCTC
MPK12_InF_pET_R	gtcgacggagctcgaattcTCAGTGGTCAGGATTGAATTTG
LongHT1_USER_F	ggcttaauATGTCTGGTTTATGTTTCAATCC
MPK4_user_R	ggtttaauTCACACTGAGTCTTGAGGATTG
MPK12_user_R	ggtttaauTCAGTGGTCAGGATTGAATTTGAC
HT1_user_R	ggtttaauTCAGGCATTTACAGGAACAGAG
HT1.pET.InF.F	gctgatatcggatccATGGAGAAGAAGAGATTTGAC
HT1.pET.InF.R	cggagctcgaattcGGCATTTACAGGAACAGAG
HT1long_pET_InF_F	gctgatatcggatccATGTCTGGTTTATGTTTCAATCC
CBC1_USER_F	ggcttaauATGAAGGAGAAGGCGGAG
CBC1_USER_R	ggtttaauTGGGCCTCGGTGTCGG
CBC2_USER_F	ggcttaauATGAAAGAAGGAAAGGATGGG
CBC2_USER_R	ggtttaauAGGACCACGTTTCCTTCG
CBC1_S280A_F	GTGGCTCGTCTAGAGGCTgCAAACCCTAACGACATGAC
CBC1_S280A_R	GTCATGTCGTTAGGGTTTGcAGCCTCTAGACGAGCCAC
CBC1_S43&45A_F	GGACGATGGAGAAGAGGAAGgcTTTGgcTGATGGTGAAGATAACG
CBC1_S43&45A_R	CGTTATCTTCACCATCAgcCAAAgcCTTCCTCTTCTCCATCGTCC
CBC1_T256A_Fw	GATTGTGCATAGAGACGTGAAAgCGGAGAACATGCTTTTAG
CBC1_T256A_Rv	CTAAAAGCATGTTCTCCGcTTTCACGTCTCTATGCACAATC
CBC1_D253A_F	CTCAGAAGATTGTGCATAGAGcCGTGAAAACGGAGAAC
CBC1_D253A_R	GTTCTCCGTTTTCACGgCTCTATGCACAATCTTCTGAG
HT1_A109A_Fw	gggatctacaaacaaagagTcgttgccgtgaagatggtg
HT1_A109A_Rv	caccatcttcacggcaacgActctttgtttgtagatccc
HT1_G89R_Fw	GATCTTTCGCAGCTATTTATCaGAAACAAATTTGCTTCCGG
HT1_G89R_Rv	CCGGAAGCAAATTTGTTTCtGATAAATAGCTGCGAAAGATC
HT1_R173Q_Fw	GTCACAAGGAAACCTCCaAATGTACCTAAACAAGAAAGAG
HT1_R173Q_Rv	CTCTTTCTTGTTTAGGTACATTtGGAGGTTTCCTTGTGAC
MPK12_Y277G_Fw	GCAGTGACAACGCAAGAAGAggCGTCAGGCAACTTCCGCG
MPK12_Y277G_Rv	CGCGGAAGTTGCCTGACGccTCTTCTTGCGTTGTCACTGC

Table S3. Primer sequences used in this study.
